# A thermodynamic description for physiological transmembrane transport

**DOI:** 10.12688/f1000research.16169.3

**Published:** 2021-05-19

**Authors:** Marco Arieli Herrera-Valdez

**Affiliations:** 1 Department of Mathematics, Facultad de Ciencias, Universidad Nacional Autonoma de Mexico, CDMX, 04510, Mexico

**Keywords:** Transmembrane transport, ion channels, passive transport, active transport, rectification, bidirectional assymetric flow, AMPA-Kainate receptor, excitable cell

## Abstract

A general formulation for both passive and active transmembrane transport is derived from basic thermodynamical principles. The derivation takes into account the energy required for the motion of molecules across membranes and includes the possibility of modeling asymmetric flow. Transmembrane currents can then be described by the general model in the case of electrogenic flow. As it is desirable in new models, it is possible to derive other well-known expressions for transmembrane currents as particular cases of the general formulation. For instance, the conductance-based formulation for current turns out to be a linear approximation of the general formula for current. Also, under suitable assumptions, other formulas for current based on electrodiffusion, like the constant field approximation by Goldman, can be recovered from the general formulation. The applicability of the general formulations is illustrated first with fits to existing data, and after, with models of transmembrane potential dynamics for pacemaking cardiocytes and neurons. The general formulations presented here provide a common ground for the biophysical study of physiological phenomena that depend on transmembrane transport.

## Introduction

One of the most important physiological mechanisms underlying communication within and between cells is the transport of molecules across membranes. Molecules can cross membranes either passively ( Stein & Litman, 2014), or via active transport ( Bennett, 1956). Molecules are passively transported across a membrane when they move along their (electro)chemical gradient. In contrast, active transport involves transmembrane motion of molecules against their electrochemical gradients. One important functional distinction between channels and pumps is that the rate of transport for channels is generally several orders of magnitude faster than the rate for pump-mediated transport ( Gadsby, 2009; Ussing, 1949a). Such differences are reflected in the sizes of different transmembrane fluxes typically observed in excitable cells ( Herrera-Valdez & Lega, 2011).

*Passive* transport may occur through transmembrane proteins ( Hille, 1992; Stein & Litman, 2014) that may be selective for molecules of specific types ( Almers & McCleskey, 1984; Doyle *et al*., 1998; Favre *et al*., 1996), typically mediating (electro)diffusion through them. Sometimes these proteins are also gated by conformational changes triggered by different signlling mechanisms. Passive transport has also been observed in pores spontaneously formed within synthetic lipid bilayers ( Blicher & Heimburg, 2013), which could also occur in natural conditions ( Gurtovenko & Anwar, 2007). One example of importance in the context of energy homeostasis is the transport of monosaccharides and other similar molecules through GLUT transporters. GLUT transporters first bind to their substrates, triggering a conformational change that allows the substrate to cross in the direction of its chemical gradient (e.g. GLUT5 is highly specific to fructose, Mueckler & Thorens (2013)). Channels are another important class of transmembrane proteins that typically mediate fast passive transport, often displaying selectivity for specific ion types, gated by changes in the membrane potential ( Covarrubias *et al*., 1991; Peng & Wu, 2007) or the binding of a ligand molecule (e.g. AMPA-Kainate synaptic receptors, Bowie (2002)).

*Active* transport is mediated by transmembrane proteins commonly called pumps, or carriers, that mechanically translocate the molecules they transport ( Bennett, 1956; Ussing, 1949b; Ussing, 1949c). The energy for primary active transport is usually obtained from biochemical reactions (e.g. ATPases, light-driven pumps). For instance, the energy to transport molecules against their (electro)chemical gradient via ATPases is obtained from hydrolysis of ATP ( Chapman, 1973). In secondary and tertiary active transport, the electrochemical gradient of at least one the ion types provides the energy to transport other molecules against their (electro)chemical gradient ( Skou, 1965). Two large classes of non-primary active transport pumps are the symporters and counterporters, carrying at least two types of molecules in the same, or in opposite directions, respectively, with at least one type against its electrochemical gradient. For instance Na-H exchangers carry Na ^^+^^ and H ^^+^^ in opposite directions, typically using the driving force from Na ^^+^^. In contrast, K-Cl symporters carry K ^^+^^ and Cl ^*−*^ in the same direction, which means that one of the two ion types is carried against its concentration gradient. This is because the K ^^+^^ and Cl ^*−*^ concentration gradients are typically oriented in opposite directions. As a consequence, the movement of one of the two ions releases energy from its electrochemical gradient, enabling the transport of the other ion against its gradient.

Theoretical models of transmembrane transport play a critical role in developing our understanding of the function and mechanisms underlying electrical signalling and cellular excitability ( Barr, 1965; Cole, 1965; DiFrancesco & Noble, 1985; Endresen *et al*., 2000; Gadsby, 2009; Goldman, 1943; Kell, 1979; Kimizuka & Koketsu, 1964; Läuger, 1973; Stevens & Tsien, 1979; Wiggins, 1985a; Wiggins, 1985b; Wiggins, 1985c), and some of its associated pathologies ( Ashcroft, 2005; Marbán, 2002). The best known transmembrane transport models include the widely used conductance-based formulation from the seminal work of Hodgkin & Huxley (1952), the Goldman-Hodgkin-Katz equation ( Goldman, 1943; Hodgkin & Katz, 1949; Pickard, 1976), and several other expressions for carrier and channel mediated transport with many different functional forms ( DiFrancesco & Noble, 1985; Rasmusson *et al*., 1990a; Rasmusson *et al*., 1990b; Rosenberg & Wilbrandt, 1955). Other formulations for *ionic* transport across membranes derived from biophysical principles available in the literature include those in the work by Jacquez & Schultz (1974); Kimizuka & Koketsu (1964); Pickard (1969); Pickard (1976). See also Jacquez (1981) and similar work by Endresen *et al*. (2000), and those in the excellent book by Johnston *et al*. (1995). Such formulations describe the relationship between the activity and permeability of ions across membranes, and the transmembrane potential. However general models that describe physiological transport including passive and active transport of charged or non-charged molecules, with bidirectional but possibly asymmetric flows, are still missing. The work presented here builds upon the results previously mentioned by describing transport macroscopically in terms of the energy required to move molecules across a membrane. The result is a general formulation with a common functional form for both passive and active transport ( Herrera-Valdez, 2014) that also includes a term that regulates the asymmetry in the flow (rectification). The details of the derivation and examples of fits to experimental data and features like asymmetric bidirectional flow can be found in the next section. An application of the general formulation is illustrated with models for the transmembrane potential dynamics in cardiac pacemaker cells and striatal fast spiking interneurons. A derivation and connection of the general formula with existing formulations like the Goldman expression for current can be in the paragraphs below.

## General formulation for transmembrane flux and current

### Work required for transmembrane molecular fluxes

Consider a system consisting of a biological membrane surrounded by two aqueous compartments (e.g. extracellular and intracellular). Assume, to start with, that the compartments contain molecules of a single type *s* ( *e.g.* Na ^^+^^, K ^^+^^, glucose), possibly in different concentrations. Let ∆ *G _s_* be the energy required for the transport of the molecules across the membrane in a specific direction (e.g. inside to outside). To write an expression for ∆ *G _s_* it is necessary to take the direction of motion of the *s*-molecules into account. To do so, label the extracellular and intracellular compartments as 0 and 1, respectively, and let *c _s_* and *d _s_* ∈ {0, 1} represent the source and the destination compartments for the transport of the *s*-molecules. The pair ( *c _s_*, *d _s_* )=(0,1) represents inward transport and the pair ( *c _s_*, *d _s_* )=(1,0) represents outward transport. The work required to transport *n _s_* molecules of type *s* from compartment *c _s_* to compartment *d _s_* can then be written as

$$\;\Delta G_{s}=n_{s}(c_{s}-d_{s})\left[kT ln\left(\frac{{\left[s\right]}_{0}}{{\left[s\right]}_{1}}\right)-\text{q}z_{s}v\right],\;(1)$$

( Aidley, 1998; Blaustein *et al*., 2004; De Weer *et al*., 1988) where T, k, q, *z _s_*, [ *s*] _0_, and [ *s*] _1_ represent the absolute temperature (°K), Boltzmann's constant (1.381 ×10 ^−23^ Joules/ ^*o*^Kelvin), the elementary charge (1.60217733 ×10 ^−19^ Coulombs/molecule), the valence, the extracellular, and the intracellular concentrations for the molecules of type *s*, respectively. The variable *v* represents the transmembrane potential. Two particular cases are worth noticing. First, if *s* is an ion, then *z _s_* ≠ 0 and Equation (1) becomes

$$\;\Delta G_{s}=\text{q}z_{s}n_{s}\left(c_{s}-d_{s}\right)\left(v_{s}-v\right),\;(2)$$

where *v _s_* is the Nernst potential for the *s*-molecules ^1^ ( Nernst, 1888). Second, if the *s*-molecules are not charged, then *z _s_* = 0 and the work required to move the *s*-type molecules from *c _s_* to *d _s_* simplifies to

$$\;\Delta G_{s}=n_{s}(c_{s}-d_{s})kT ln\left(\frac{{\left[s\right]}_{0}}{{\left[s\right]}_{1}}\right).\;(3)$$

If ∆ *G _s_* < 0, then the molecules can be transported passively ( *e.g.* electrodiffusion), decreasing the electrochemical gradient for *s* across the membrane. In contrast, if ∆ *G _s_* > 0, the transmembrane transport of *s* from *c _s_* to *d _s_* is not thermodynamically favourable, which means the transport from *c _s_* to *d _s_* requires energy that is not available in the electrochemical gradient for *s* (active transport). As a consequence, active transport of *s* would increase the driving force for the motion of *s* across the membrane.

### Joint transmembrane transport of different types of molecules

To find an expression that describes a more general transport mechanism, assume that transport takes place as single events in which molecules of *m* different types move in parallel, or possibly sequentially (e.g. first Na ^^+^^, then K ^^+^^), across the membrane. Let *S* be a set that represents the types of molecules that are jointly transported in a single event. For instance, for Na ^^+^^-H ^^+^^ exchangers, *S* = {Na ^^+^^, H ^^+^^}, with *m* = 2. The energy required to transport these molecules is the sum of the energies required to transport each type of molecule in *S*. In other words,

$$\;\Delta G_{S}=\sum_{s\in S}\Delta G_{s}.\;(4)$$

As before, transport is thermodynamically favourable when ∆ *G _S_* ≤ 0. If not, extra energy is required. To distinguish between these two cases, define the total energy ∆ *G* of the transport mechanism, possibly including an extra source of energy, as

$$\;\Delta G=\delta_{\text{Ext}}\Delta G_{\text{Ext}}+\;\Delta G_{S},\;(5)$$

where *δ*_Ext_ = 1 if ∆ *G _S_* > 0, and 0 otherwise. If ∆ *G _S_ >* 0, then the energy from ATP hydrolysis or any other sources represented by *δ*_Ext_∆ *G*_Ext_ should be negative and larger in size in comparison to ∆ *G _S_*, so that ∆G ≤ 0, making the transport thermodynamically possible. In particular, for ATP-driven transport, the extra energy supplied by hydrolysis of ATP ( Tanford, 1981; De Weer *et al*., 1988) is

$$\;\Delta G_{\text{ATP}}=\Delta G_{\text{ATP}}^{0}+\text{kT ln}\left(\frac{{\text{[ADP]}}_{\text{1}}{{\text{[P}}_{i}\text{]}}_{\text{1}}}{{\text{[ATP]}}_{\text{1}}}\right)=\text{q}v_{\text{ATP}},\;(6)$$

with [ATP] _1_, [ADP] _1_, and [P *_i_*] _1_ representing, respectively, the intracellular concentrations of adenosine triphosphate (ATP), adenosine diphosphate (ADP), and inorganic phosphate (P *_i_*) ( De Weer *et al*., 1988). The potential *v*_ATP_ ≈ −450 mV ( Endresen *et al*., 2000), but could vary depending on the intracellular amounts of ATP, ADP, and P *_i_* ( De Weer *et al*., 1988). The first term in Equation (6) represents the "standard" change in Gibbs free energy for the hydrolysis of ATP ^2^. Similar expressions could be derived for active transport driven by light, or other sources of energy. The concentrations of ATP, ADP, and P *_i_* are assumed to be constant in most models presented here, but it should be noted that such concentrations are not necessarily constant, and in fact, may vary a lot in some cases, as it is the case for skeletal muscle ( Wackerhage *et al*., 1998).

### Flux due to transmembrane transport

The formulation in Equation (5) can be combined with Equation (1) to derive a generalised expression for flux and model different known mechanisms of physiological transmembrane transport, possibly combining the transport of different molecules simultaneously (e.g. Na-H exchange). In this case, the forward direction of the transport would be described by the combined forward transport of each of the different molecules under consideration. For instance, the source and target compartments for Na ^+^ and Ca ^2+^ are different in Na-Ca exchangers. The stoichiometry for the transport mediated by Na-Ca exchangers in the forward direction involves three Na ^+^ molecules moving inward (along their electrochemical gradient) in exchange for one Ca ^2+^ molecule moving outward (against their electrochemical gradient) ( Mullins, 1979; Venetucci *et al*., 2007).

Let *α* and *β* be the flux rates in the forward and backward directions, in units of molecules per ms per *µ*m ^*−*2^. These rates depend, *a priori*, on the energy required for the transport of the molecules in *S*. The net *flux* rate associated to the net transmembrane transport, can then be written as

Φ(ΔG)=α(ΔG) –β(ΔG).(7)

How do *α* and *β* depend on ∆ *G*? The steady state relationship between the energy ∆ *G* and the the forward and backward flow rates, hereby represented by *α* and *β*, can be expressed by means of a Boltzmann distribution ( Boltzmann, 1868) as

$$\;\frac{\alpha }{\beta }(\Delta G)=\exp\left(-\frac{\Delta G}{\text{kT}}\right).\;(8)$$

**Figure 1.**
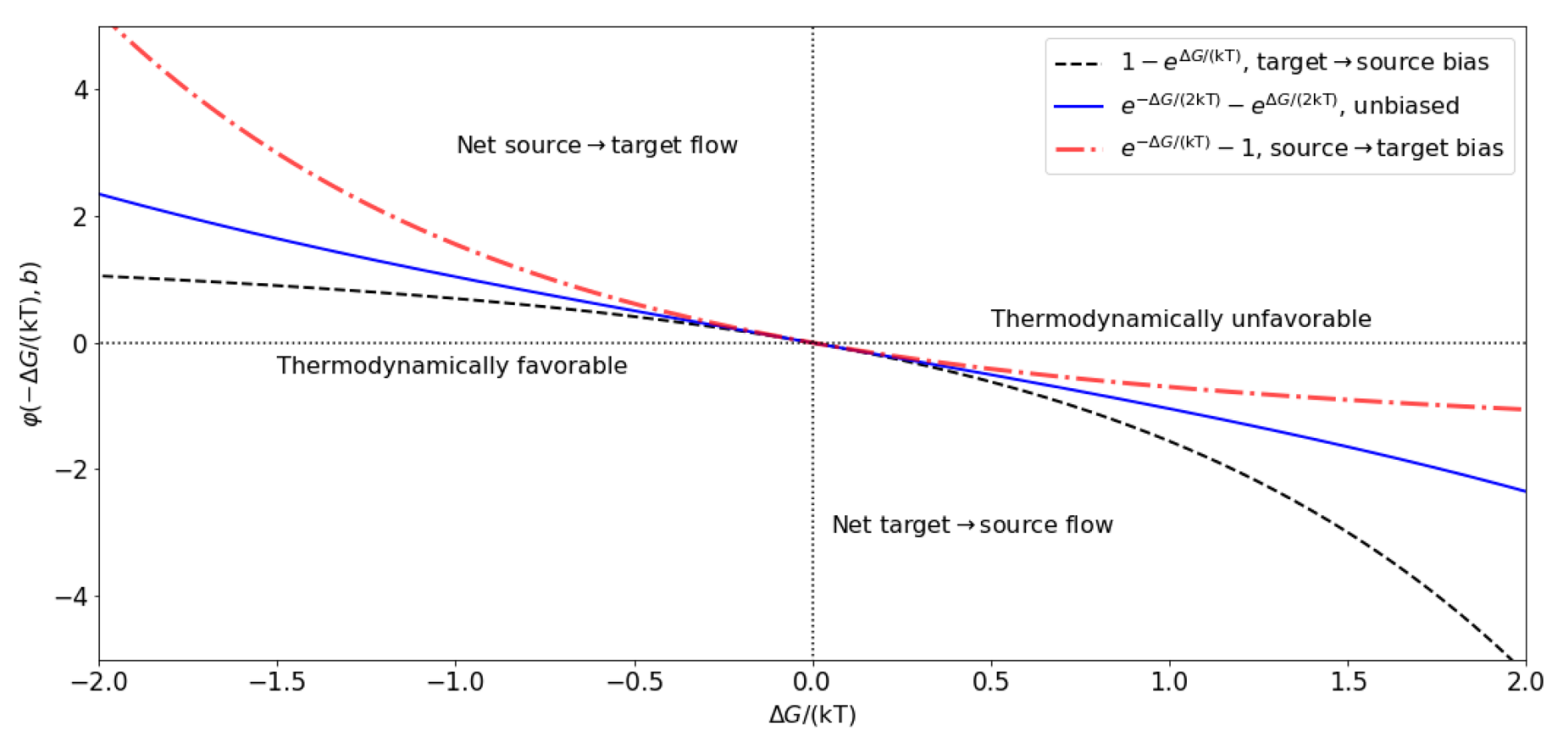
Fluxes biased in the target *→*source (backward, *b*=0.1, black dashed line), source *→*target (forward, *b*=0.9, red dash-dot line), or showing no rectification ( *b*=0.5, blue solid line). The energy ∆ *G _S_* necessary can be found on the right axis if the transport is primary active, and on the left axis when transport is secondary active or passive. Extra energy from ATP hydrolysis or other sources added to ∆ *G _S_* shift the total energy to the left axis and make the transport thermodynamically possible. See Table 1 for examples.

Assuming that *α* and *β* are continuous functions, the rates *α* and *β* can be rewritten as

$$\;\alpha =r\exp\left(-b\frac{\Delta G}{\text{kT}}\right),\;\beta =r\exp\left((1-b)\frac{\Delta G}{\text{kT}}\right),\;(9)$$

where *r* and *b* represent the rate at which the transport takes place (molecules per ms per *µ*m ^*−*2^) and the bias of the transport in one of the two directions ( *b* = 1 */*2 means the transport is symmetrical relative to the point at which ∆ *G*=0). Note that the functional form of the *α* and *β* in Equations (9) are similar to those by Butler (1924); Erdey-Grúz & Volmer (1930). Also, notice that the steady state relationship between *α* and *β* in Equation (8) can be obtained from Equations (9), for any *r* and any *b*. However, it should be the case that *r* and *b* vary in specific ranges depending on the physicochemical characteristics of the pore through which molecules cross the membrane, and in general, on the transport mechanism. As already mentioned, the rate *r* should be larger for electrodiffusive transport mediated by ion channels, in comparison to the slower transport rates for facilitated diffusion and active transport meditated by carrier proteins and pumps. The rate *r* may depend on temperature ( Sen & Widdas, 1962), the transmembrane potential ( Starace *et al*., 1997), the concentrations inside and outside of the membrane ( Yue *et al*., 1990), and other factors ( Novák & Tyson, 2008). If the parameter *b* ∈ [0, 1], then *b*∆ *G* and ( *b −* 1)∆ *G* have opposite signs and can be thought of as the energies required to the transport of the molecules in *S* in the forward and backward directions, respectively, with *b* biasing the transport in the forward direction when close to 1, and in the backward direction when close to 0 ( Figure 1).

**Table 1.** Energy required for transmembrane transport mediated by different passive and active mechanisms.

Pump or channel	Molecule (s)	*n _s_*	*c _s_*	*d _s_*	*c _s_*– *d _s_*	$\Delta G_{s}=\text{q}z_{s}n_{s}(c_{s}-d_{s})(v_{s}-v)$	*η*	*v _o_*	α/ *β* = exp $\left(-\frac{\Delta G}{\text{kT}}\right)$
Cl ^–^ channel	Cl ^–^	1	0	1	-1	Δ *G*_Cl_ = q( *v*_Cl_ – *v*)	1	*v*_Cl_	$\left(\frac{{\left[Cl\right]}_{0}}{{\left[Cl\right]}_{1}}\right)\exp\left(\frac{v}{v_{\text{T}}}\right)$
K ^+^ channel	K ^+^	1	1	0	1	Δ *G*_K_ = q( *v*_K_ – *v*)	1	*v*_K_	$\left(\frac{{\left[\text{K}\right]}_{1}}{{\left[\text{K}\right]}_{0}}\right)\exp\left(\frac{v}{v_{\text{T}}}\right)$
Na ^+^ channel	Na ^+^	1	0	1	-1	Δ *G*_Na_ = –q( *v*_Na_ – *v*)	-1	– *v*_Na_	$\left(\frac{{\left[Na\right]}_{0}}{{\left[Na\right]}_{1}}\right)\exp\left(-\frac{v}{v_{\text{T}}}\right)$
Ca ^2+^ channel	Ca ^2+^	1	0	1	-1	Δ *G*_Ca_ = –2q( *v*_Ca_ – *v*)	-2	–2 *v*_Ca_	$\left(\frac{{\left[Ca\right]}_{0}}{{\left[Ca\right]}_{1}}\right)\exp\left(-2\frac{v}{v_{\text{T}}}\right)$
Na ^+^-K ^+^ ATPase	Na ^+^ K ^+^	3 2	1 0	0 1	1 -1	Δ *G*_Na_ = 3q( *v*_Na_ – *v*) Δ *G*_K_ = –2q( *v*_K_ – *v*)	1	*v*_ATP_ + 3 *v*_Na_ – 2 *v*_K_	${\left(\frac{{\left[\text{Na}\right]}_{1}}{{\left[\text{Na}\right]}_{0}}\right)}^{3}{\left(\frac{{\left[\text{K}\right]}_{0}}{{\left[\text{K}\right]}_{1}}\right)}^{2}\exp\left(\frac{v-v_{\text{ATP}}}{v_{\text{T}}}\right)$
Ca ^2+^ ATPase	Ca ^2+^	1	1	0	1	Δ *G*_Ca_ = 2q( *v*_Ca_ – *v*)	2	*v*_ATP_ + 2 *v*_Ca_	$\left(\frac{{\left[Ca\right]}_{1}}{{\left[Ca\right]}_{0}}\right)\exp\left(\frac{2v-v_{\text{ATP}}}{v_{\text{T}}}\right)$
H ^+^ ATPase	H ^+^	1	1	0	1	Δ *G*_H_ = q( *v*_H_ – *v*)	1	*v*_ATP_ + *v*_H_	$\left(\frac{{\left[H\right]}_{1}}{{\left[H\right]}_{0}}\right)\exp\left(\frac{v-v_{\text{ATP}}}{v_{\text{T}}}\right)$
Na ^+^-Ca ^2+^ exchanger	Na ^+^ Ca ^2+^	3 1	0 1	1 0	-1 1	Δ *G*_Na_ = –3q( *v*_Na_ – *v*) Δ *G*_Ca_ = 2q( *v*_Ca_ – *v*)	-1	2 *v*_Ca_ – 3 *v*_Na_	${\left(\frac{{\left[\text{Na}\right]}_{0}}{{\left[\text{Na}\right]}_{1}}\right)}^{3}\left(\frac{{\left[\text{Ca}\right]}_{1}}{{\left[\text{Ca}\right]}_{0}}\right)\exp\left(-\frac{v}{v_{\text{T}}}\right)$
Na ^+^-I ^–^ symporter	Na ^+^ I ^–1^	2 1	0 0	1 1	-1 -1	Δ *G*_Na_ = –2q( *v*_Na_ – *v*) Δ *G*_1_ = –q( *v*_1_ – *v*)	-1	– *v*_1_ – 2 *v*_Na_	${\left(\frac{{\left[\text{Na}\right]}_{0}}{{\left[\text{Na}\right]}_{1}}\right)}^{2}\left(\frac{{\left[\text{I}\right]}_{0}}{{\left[\text{I}\right]}_{1}}\right)\exp\left(-\frac{v}{v_{\text{T}}}\right)$
Na ^+^-H ^+^ exchanger	Na ^+^ H ^+^	1 1	0 1	1 0	-1 1	Δ *G*_Na_ = –q( *v*_Na_ – *v*) Δ *G*_H_ = q( *v*_H_ – *v*)	0	*v*_H_ – *v*_Na_	$\left(\frac{{\left[\text{H}\right]}_{1}}{{\left[\text{H}\right]}_{0}}\right)\left(\frac{{\left[\text{Na}\right]}_{0}}{{\left[\text{Na}\right]}_{1}}\right)$
K ^+^-Cl ^–^ symporter	K ^+^ Cl ^–^	1 1	1 1	0 0	1 1	Δ *G*_K_ = q( *v*_K_ – *v*) Δ *G*_Cl_ = –q( *v*_Cl_ – *v*)	0	*v*_K_ – *v*_Cl_	$\left(\frac{{\left[\text{K}\right]}_{1}}{{\left[\text{K}\right]}_{0}}\right)\left(\frac{{\left[\text{Cl}\right]}_{1}}{{\left[\text{Cl}\right]}_{0}}\right)$
Na ^+^-K ^+^-Cl ^–^ symporter	Na ^+^ K ^+^ Cl ^–^	1 1 2	0 0 0	1 1 1	-1 -1 -1	Δ *G*_Na_ = –q( *v*_Na_ – *v*) Δ *G*_K_ = –q( *v*_K_ – *v*) Δ *G*_Cl_ = 2q( *v*_Cl_ – *v*)	0	2 *v*_Cl_ – *v*_Na_ – *v*_K_	$\left(\frac{{\left[\text{Na}\right]}_{0}}{{\left[\text{Na}\right]}_{1}}\right)\left(\frac{{\left[\text{K}\right]}_{0}}{{\left[\text{K}\right]}_{1}}\right){\left(\frac{{\left[\text{Cl}\right]}_{0}}{{\left[\text{Cl}\right]}_{1}}\right)}^{2}$

The flux can then be written explicitly combining Equation (7) and Equation (9) to obtain,

$$\;\text{Φ}(\Delta G)=r\left[\exp\left(-b\frac{\Delta G}{\text{kT}}\right)-\exp\left((1-b)\frac{\Delta G}{\text{kT}}\right)\right].\;(10)$$

Taking the above observations into account, it is possible to combine Equation (4) and Equation (5), to write an expression similar to Equation (8) for the steady state balance between the forward and backward transport of all the molecules in *S* (see Table 1 for examples of different transport mechanisms with their energies and total charge movements).

### Flux and current

After substitution of the formulas for ∆ *G* from Equation (4) and Equation (5) into Equation (10), the flux rate resulting from simultaneously transporting molecules in *S* across the membrane can be written explicitly as

$$\Phi =r\left[\prod_{s\in S}{\left(\frac{{\left[s\right]}_{0}}{{\left[s\right]}_{1}}\right)}^{bn_{s}(d_{s}-c_{s})}\exp\left(b\frac{\eta v-\delta_{\text{Ext}}v_{\text{Ext}}}{v_{\text{T}}}\right)-\prod_{s\in S}{\left(\frac{{\left[s\right]}_{0}}{{\left[s\right]}_{1}}\right)}^{\left(b-1)n_{s}(d_{s}-c_{s}\right)}\exp\left((b-1)\frac{\eta v-\delta_{\text{Ext}}v_{\text{Ext}}}{v_{\text{T}}}\right)\right],\;(11)$$

where *v*_T_ = kT */*q and

$$\;\;\eta =\sum_{s\in S}n_{s}(c_{s}-d_{s})z_{s},\;(12)$$

represents the net number of charges moved across the membrane. Note that *v*_Ext_ should be *v*_ATP_ for ATPases. The first, more complex, form of the flux in Equation (11) could be useful when working with models for which changes in the concentrations of different molecules are relevant.

If the transport is electrogenic, then the product q *η* (in Coulombs) represents the net charge moved across the membrane, relative to the extracellular compartment. Non electrogenic transport yields *η* = 0, which means the flow does not depend on the transmembrane potential, and

$$\Phi =r\left[\prod_{s\in S}{\left(\frac{{\left[s\right]}_{0}}{{\left[s\right]}_{1}}\right)}^{bn_{s}(d_{s}-c_{s})}\exp\left(-b\frac{\delta_{\text{Ext}}v_{\text{Ext}}}{v_{\text{T}}}\right)-\prod_{s\in S}{\left(\frac{{\left[s\right]}_{0}}{{\left[s\right]}_{1}}\right)}^{\left(b-1)n_{s}(d_{s}-c_{s}\right)}\exp\left((1-b)\frac{\delta_{\text{Ext}}v_{\text{Ext}}}{v_{\text{T}}}\right)\right].\;(13)$$

If only ions are involved in the transport, the flux simplifies to

$$\;\Phi =r\left\{\exp\left[b\left(\frac{\eta v-v_{o}}{v_{\text{T}}}\right)\right]-\exp\left[(b-1)\left(\frac{\eta v-v_{o}}{v_{\text{T}}}\right)\right]\right\},\;(14)$$

where

$$\;v_{o}=\delta_{\text{Ext}}v_{\text{Ext}}+\sum_{s\in S}n_{s}z_{s}(c_{s}-d_{s})v_{s}.\;(15)$$

The quantity *v _o_/η* can be thought of as a reversal potential. If *η <* 0, then positive charge is transported inward, or negative charge is transported outward. In contrast, *η >* 0 means that positive charge is transported outward or negative charge transported inward. For instance, inward electrodiffusion of single Na ^+^ ions gives *η* = *−*1, which can be thought of as loosing one positive charge from the extracellular compartment in each transport event.

**Transmembrane current.** A flux that results in electrogenic transport ( Equation (11) and Equation (14)) can be converted to current density after multiplication by q *η*. In short form,

i=qηΦ(16)

with q *r* in Amperes/m ^2^ or equivalent units.

**Figure 2.**
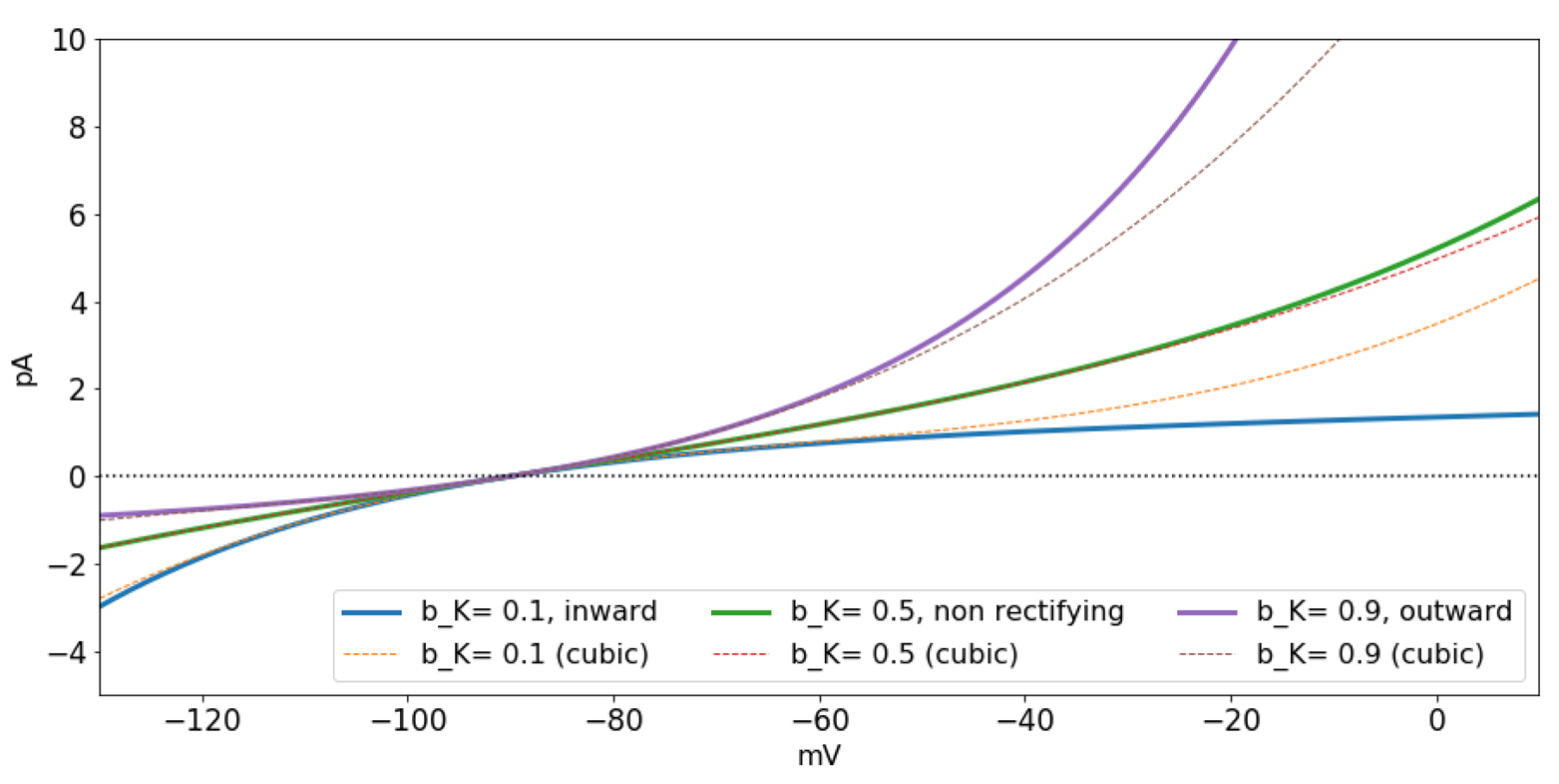
Currents mediated by K-electrodiffusion for *b*_K_ ∈ {0.1, 0.5, 0.9} using the general formulation (solid lines) and its cubic approximation (dashed lines). Inward rectification occurs for *b _K_* < 1 */*2 and outward rectification for *b*_K_ > 1 */*2 and q *r*_K_ *N*_K_ = 1.

Substitution of Equation (11) or Equation (14) into Equation (16) yields a general formula for the current generated by transmembrane ionic flux ( Figure 2), that uses the same functional form for channels (protein or lipid) and pumps. Recall that Equation (16) can also be written explicitly in terms of the transmembrane concentrations of one or more of the ions involved using Equation (11). It is possible to derive expressions for *r* that take into account biophysical variables like temperature and the shape and length of the pore through which the molecules cross ( Endresen *et al*., 2000; Pickard, 1969).

### Special cases and examples

A number of nontrivial and important properties of transmembrane ionic currents, including rectification, are also described by Equation (16). Also, different models for current already in the literature can be obtained by making approximations or setting particular cases from Equation (16). Examples include electrodiffusive currents that result from integration of the Nernst-Planck equation along the length of membrane pore ( Jacquez & Schultz, 1974; Johnston *et al*., 1995; Pickard, 1969). Of particular interest, conductance-based currents are linear approximations of the formulation ( 16), around the reversal potential for the current.

**Lower order approximations to the general formulation and conductance based models.** The general current from Equation (16) can be rewritten as a series around the reversal potential *v _o_* to obtain useful approximations with possible computational or analytical advantages. To do so, rewrite equation Equation (16) using Taylor’s theorem ( Courant & John, 2012; Spivak, 2018) to obtain

$$\;i=\text{q}\eta r\left[\left(\frac{\eta v-v_{o}}{v_{\text{T}}}\right)+\left(b-\frac{1}{2}\right){\left(\frac{\eta v-v_{o}}{v_{\text{T}}}\right)}^{2}+\left(\frac{3b^{2}-3b+1}{3!}\right){\left(\frac{\eta v-v_{o}}{v_{\text{T}}}\right)}^{3}+\ldots \right].\;(17)$$

Conductance-based currents ( Hodgkin & Huxley, 1952) can then be obtained as linear approximations of the general current around *v _o_*,

$$\;i\approx g\left(v-\frac{v_{o}}{\eta }\right),\;(18)$$

where *g* = *η* ^2^q *r/v*_T_ is in units of nS/ *µ*m ^2^. For instance, the linear approximation for the current through open sodium channels around *v*_Na_ in Equation (18) gives *g*_Na_ = q *r*_Na_ */v*_T_, and *v _o_*/ *η*_Na_ = *v*_Na_, with *η*_Na_ = *−*1, so that *i*_Na_ ≈ g _Na_( *v − v*_Na_).

Note that if the truncation order is larger than 1, then approximations from Equation (17) include the rectification parameter *b*. One already known consequence is that conductance-based currents do not capture rectification.

**The Goldman constant field approximation is a particular case of the general formulation.** The Goldman-Hodgkin-Katz equation describing the transmembrane current carried by ions of type *x* is given by

$$\;I_{x}=g_{x}\;v\left[\frac{x_{0}-x_{1}\exp\left(z_{x}\frac{v}{v_{T}}\right)}{1-\exp\left(z_{x}\frac{v}{v_{T}}\right)}\right],\;(19)$$

where *x _j_*, *j* ∈ {0, 1}, represent the extra and intracellular concentrations of an ion of type *x*.

Multiplying the numerator and denominator of Equation (19) by $x_{1}^{b-1}x_{0}^{b}\exp[(b-1)z_{x}v/v_{T}]$, and algebraically rearranging terms yields,

$$\;I_{x}=A_{x}\left[\exp\left(bz_{x}\frac{v-v_{x}}{v_{T}}\right)-\exp\left((b-1)z_{x}\frac{v-v_{x}}{v_{T}}\right)\right],\;(20)$$

where

$$\;A_{x}=\frac{g\;v\;x_{1}^{1-b}\;x_{0}^{b}}{\exp\left(bz_{x}\frac{v}{v_{T}}\right)-\exp\left((b-1)z_{x}\frac{v}{v_{T}}\right)},\;(21)$$

is an amplitude term that can be approximated by a constant ( Endresen *et al.*, 2000; Herrera-Valdez & Lega, 2011; Nonner & Eisenberg, 1998; Nonner *et al.*, 1998, Equation 25 and Equation 26).

**Asymmetric bidirectional flow causes rectification.** If the flux of molecules across a membrane is mediated by proteins, it can be biased in either the outward or the inward direction ( Hollmann *et al.*, 1991; Riedelsberger *et al.*, 2015). This was first called ”anomalous rectification” by Katz (1949), who noticed that K ^+^ flows through muscle membranes more easily in the inward, than in the outward direction ( Adrian, 1969; Armstrong & Binstock, 1965). It was later found that some K ^+^ channels display the bias in the opposite direction ( Woodbury, 1971). The former type of K ^+^ current rectification is called inward, and the latter outward ( Figure 2).

Rectification is thus a bias in one of the two directions of transport. The type of rectification (inward or outward) depends on the molecules being transported and on the structure of the proteins mediating the transport. Flux rectification is not only displayed by ions, as shown by molecules like glucose, which may cross membranes via GLUT transporters bidirectionally and asymmetrically, even if the glucose concentration is balanced across the membrane ( Lowe & Walmsley, 1986).

Rectification can be described by setting *b* in Equation (11) to values different from 1/2 and it becomes more pronounced as *b* is closer to 0 or 1. These values represent biases in the transport toward the source, or the target compartment, respectively. As a consequence, rectification yields an asymmetry in the graph of *α − β* as a function of ∆ *G* ( Figure 1). For electrogenic transport, rectification can be thought of as an asymmetric relationship between current flow and voltage, with respect to the reversal potential *v _o_*. The particular case *b* = 1 */*2 (non rectifying) yields a functional form for current similar to that proposed by Pickard (1969), and later reproduced by ( Endresen *et al*., 2000), namely

$$\;i=2\text{q}\eta r\;\sinh\left(\frac{\eta v-v_{o}}{2v_{\text{T}}}\right).\;(22)$$

From here on, subscripts will be used to represent different transport mechanisms. For instance, the current for a Na-Ca pump will be written as *i*_NaCa_.

Electrodiffusion of K ^+^ through channels ( *η* = 1 and *v _o_* = *v*_K_), is outward for *v > v*_K_, and inward for *v < v*_K_. The K ^+^ current through the open pore is therefore

$$\;i_{\text{K}}=\text{q}r_{\text{K}}\left\{\exp\left[b_{\text{K}}\left(\frac{v-v_{K}}{v_{\text{T}}}\right)\right]-\exp\left[(b_{\text{K}}-1)\left(\frac{v-v_{K}}{v_{\text{T}}}\right)\right]\right\}.\;(23)$$

Current flow through inward rectifier K-channels ( Riedelsberger *et al*., 2015) can be fit to values of *b*_K_ *<* 1 */*2. For instance,

$$\;i_{\text{K}in}=\text{q}r_{\text{K}}\left[1-\exp\left(\frac{v_{\text{K}}-v}{v_{\text{T}}}\right)\right],\;(b_{\text{K}}=0),\;(24)$$

describes a current generated by a K ^+^ flow that is limited in the outward direction, similar to the currents described originally by Katz (1949). Analogously, *b*_K_ *>* 1 */*2 limits the inward flow. For example, the current

$$\;i_{\text{K}out}=\text{q}r_{\text{K}}\left[\exp\left(\frac{v-v_{\text{K}}}{v_{\text{T}}}\right)-1\right],\;(b_{\text{K}}=1),\;(25)$$

describes outward rectification ( Riedelsberger *et al*., 2015).

Based on the work of Riedelsberger *et al*. (2015) on K ^+^ channels, inward (outward) rectification arises when the S4 segment in K ^+^ channels is located in the inner (outer) portion of the membrane. These two general configurations can be thought of in terms of ranges for the parameter *b*_K_, namely, *b*_K_ *<* 1 */*2 for inward, and *b*_K_ *>* 1 */*2 for outward rectification ( Figure 2).

**Figure 3.**
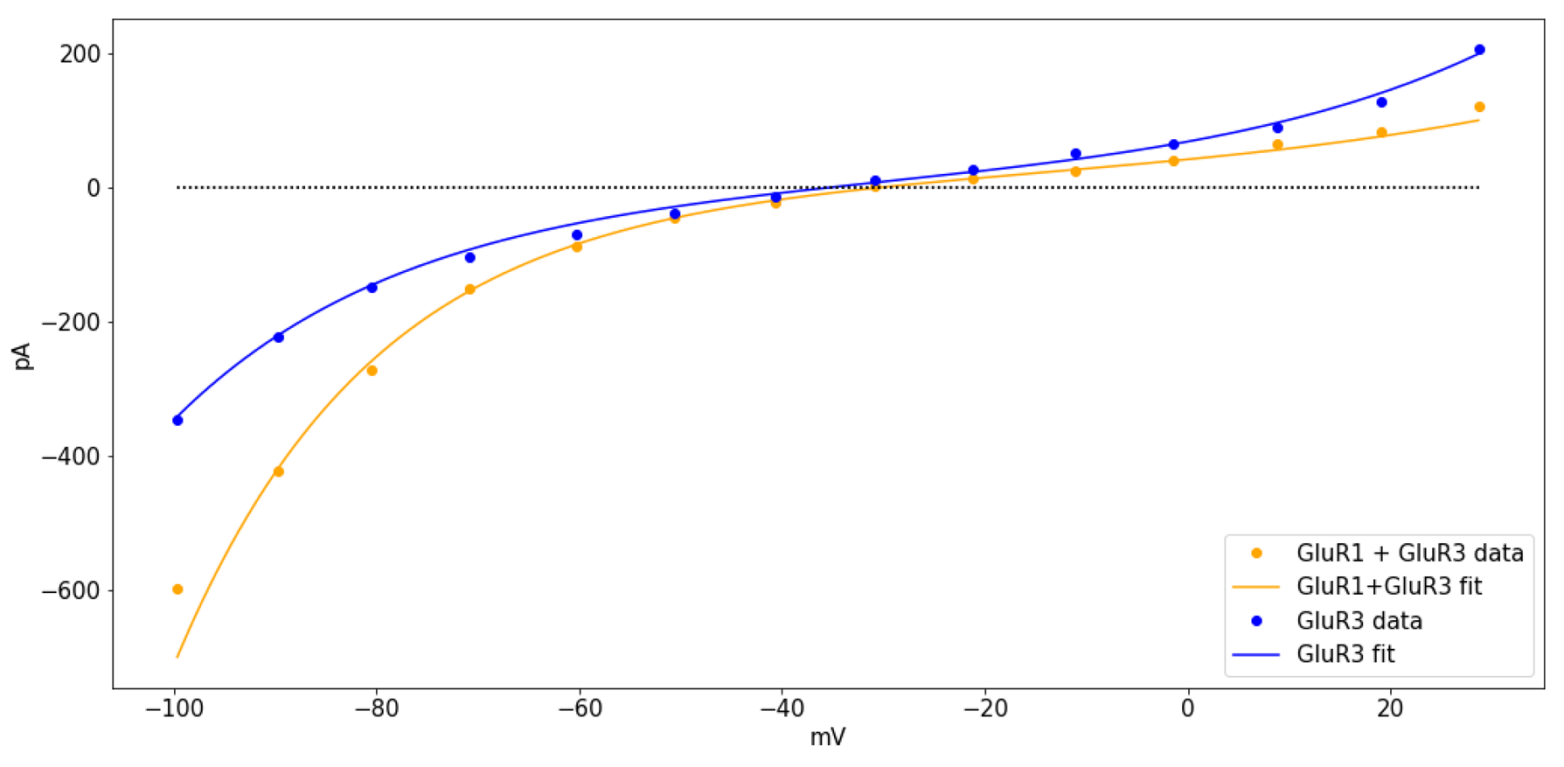
Currents mediated by AMPA-Kainate receptors with different ionic permeabilities (different reversal potentials) and different degrees of rectification caused by differences in the sub-unit composition of the receptors. Currents were recorded from oocytes injected with GluR3 cRNA (blue), or a combination of GluR1 and GluR3 cRNA (orange), in Ca ^2+^ ringer solution after activation by AMPA. The curves were fit with( *v _o_*, *b*, *r*q) = ( *−*30, 0.45, 21), for GluR3 and ( *v _o_*, *b*, *r*q) = ( *−*35, 0.35, 20), for GluR1+GluR3. Data was digitised from Figure 3B in the article by Hollmann *et al.* (1991) ( Table 2).

In general, ion channels are typically formed by different subunits, that combined produce structural changes that may result in rectified flows. For instance, non-NMDA glutamatergic receptors that can be activated by kainic acid and *α*-amino-3-hydroxy-5-methyl-4-isoxazole propionic acid (AMPA), display different permeabilities to Na ^+^, K ^+^, and Ca ^2+^ depending on the subunits that form the receptor ( Hollmann *et al*., 1991). In particular, the Ca ^2+^ currents recorded in oocytes injected with combinations of GluR1 and GluR3 cRNA have different steady state amplitudes and show different levels of rectification ( Figure 3).

**Table 2.** Current-voltage relationships for AMPA-Kainate receptors composed of different subunits. The data was digitized from Figure 3B in the article by Hollmann *et al.* (1991), using the *ginput* function from the python module matplotlib ( Hunter, 2007).

*v*-command (mV)	GluR1+GluR3	GluR3
mV	pA	pA
-99.6354	-597.802	-347.253
-89.7348	-423.077	-223.077
-80.453	-272.527	-148.352
-70.8619	-151.648	-104.396
-60.3425	-87.9121	-69.2308
-50.5967	-45.0549	-38.4615
-40.6961	-21.978	-14.2857
-30.7956	2.1978	9.89011
-21.2044	12.0879	27.4725
-11.1492	25.2747	50.5495
-1.40331	39.5604	64.8352
8.80663	63.7363	89.011
19.0166	83.5165	128.571
28.7624	119.78	205.495

**Primary active transport.** The **Na-K ATPase** is a primary active transporter that uses the energy from the hydrolysis of one molecule of ATP for the uphill transport of 3 Na ^+^ ions outward and 2 K ^+^ ions inward in each cycle ( *η*_NaK_ = 1), respectively ( De Weer *et al*., 1988). The reversal potential *v*_NaK_ = *v*_ATP_ + 3 *v*_Na_ *−* 2 *v*_K_ ( Table 1), in a single transport event ( Chapman, 1973; Gadsby *et al*., 1985; Garrahan & Glynn, 1967; Post & Jolly, 1957). As a consequence, the transport kinetics of the Na-K ATPase reverse for potentials smaller than *v*_NaK_ ( De Weer *et al*., 1988).

The current-voltage relationships recorded from Na-K ATPases in guinea pig ventricular cells are shaped as hyperbolic sines ( Gadsby *et al*., 1985). Those currents would be fit with *b*_NaK_ ≈1/2, yielding

$$\;i_{\text{NaK}}(v)=2\text{q}r_{\text{NaK}}\;\sinh\left(\frac{v-v_{\text{NaK}}}{2v_{T}}\right).\;(26)$$

In response to steroids like strophandin, the voltage-dependence of the Na-K ATPase current has been reported to show a plateau as v increases past the reversal potential for the current ( Nakao & Gadsby, 1989). In such cases, the Na-K ATPase current can be assumed to be inwardly rectifying and fit with values of *b*_NaK_ *≈* 0, so that,

$$\;i_{\text{NaK}}(v)=\text{q}r_{\text{NaK}}\left[1-\exp\left(\frac{v_{\text{ATP}}+3v_{\text{Na}}-2v_{\text{K}}-v}{v_{\text{T}}}\right)\right],\;(27)$$

or alternatively,

$$\;i_{\text{NaK}}(v)=\text{q}r_{\text{NaK}}\left[1-{\left(\frac{{\left[\text{Na}\right]}_{0}}{{\left[\text{Na}\right]}_{1}}\right)}^{3}{\left(\frac{{\left[\text{K}\right]}_{1}}{{\left[\text{K}\right]}_{0}}\right)}^{2}\exp\left(\frac{v_{\text{ATP}}-v}{v_{\text{T}}}\right)\right].\;(28)$$

The rectification for the Na-K pump ATPase has also been reported to occur in small neurons of the dorsal root ganglion in rats ( Hamada *et al*., 2003). The alternative expression ( 28) also explains qualitatively different behaviors of the Na-K current as a function of the transmembrane concentrations of Na ^+^ and K ^+^. For instance, if either [Na] _1_ or [K] _0_ increase and *v > v*_NaK_, then the amplitude of *i*_NaK_ would increase at a smaller rate of change in comparison to when *v < v*_NaK_, which grows exponentially in size. This is also in line with reports of non significant changes in the transport by Na-K ATPases in response to elevated intracellular Na ^+^ during heart failure ( Despa *et al*., 2002), in which the transmembrane potential is likely to be depolarised.

**Secondary active transport.** An example of a pump that mediates secondary active transport is the *Na-Ca exchanger*, which takes 3 Na ^+^ ions from the extracellular compartment in exchange for one intracellular Ca ^2+^ ion in forward mode ( Pitts, 1979; Reeves & Hale, 1984). The reversal potential for the current is *v*_NaCa_ = 2 *v*_Ca_ *−* 3 *v*_Na_, with *η*_NaCa_ = −1. Assuming *b*_NaCa_ = 1 */*2, the Na-Ca current can be rewritten as

$$\;i_{\text{NaCa}}(v)=2\text{q}r_{\text{NaCa}}\;\sinh(\frac{v-v_{\text{NaCa}}}{2v_{T}}).\;(29)$$

The driving force *v − v*_NaCa_ could reverse in sign with large enough increases in the intracellular concentration of Ca ^2+^, or in the membrane potential. As a result, the current could have a dual contribution to the change in transmembrane potential, as predicted by some theoretical models of cardiac pacemaker activity ( Rasmusson *et al*., 1990a; Rasmusson *et al*., 1990b).

**Electrodiffusive transport.** Consider transmembrane electrodiffusive transport of a single ionic type *x*, with *z _x_* and *v _x_* representing the valence and the Nernst potential for ions of type *x*, respectively. In this case, the reversal potential satisfies

$$v_{o}=\;n_{x}(c_{x}\;–\;d_{x})z_{x}v_{x}\;=\;\eta_{x}v_{x},\;$$

which means that *η _x_* can be factorised in the argument for the exponential functions and the general expression ( 16) can be rewritten as

$$\;i_{x}(v)=\text{q}\eta_{x}r_{x}\left\{\exp\left[\eta_{x}b_{x}\left(\frac{v-v_{x}}{v_{T}}\right)\right]-\exp\left[\eta_{x}(b_{x}-1)\left(\frac{v-v_{x}}{v_{T}}\right)\right]\right\}.\;(30)$$

In the absence of rectification ( *b _x_* = 0.5) the formula simplifies to

$$\;i_{x}(v)=2\text{q}r_{x}\sinh\left(\frac{v-v_{x}}{2v_{T}}\right).\;(31)$$

since hyperbolic sines change signs if their arguments do. For calcium channels,

$$\;i_{\text{Ca}}(v)=4\text{q}r_{\text{Ca}}\sinh\left(\frac{v-v_{\text{Ca}}}{v_{T}}\right).\;(32)$$

See Jacquez & Schultz (1974); Pickard (1969) Table 1 for other examples.

The applicability of the general formulations described above is illustrated next with models of cardiac and neuronal membrane potential.

## Transmembrane potential dynamics

To illustrate applications for the formulations discussed earlier, let us build a general model of transmembrane potential dynamics. For simplification purposes, consider only one such mechanism, labelled as *l* ∈ {1, ..., *M*}, with *p _l_N _l_* active sites (e.g. channels), where *N _l_* is the number of membrane sites where the proteins mediating the *l*th transport mechanism are found, and *p _l_* is the proportion of active sites (activation might depend on voltage or a ligand). Let *η _l_*, and *r _l_* respectively represent the net number of charges moved in each transport event, and the rate of molecular transport per unit area (molecs ms ^–1^ *⋅* *μ*m ^–2^) of a single protein mediating the *l*th current, *l* ∈ {1, ..., *M*}. Then the product *η _l_* *r _l_*q represents the current density of a single protein of type *l* (Coulombs/sec *μ*m ^2^), and the total current mediated by the *l*th mechanism in a patch of membrane can be written as *ā* *_l_ p _l_ φ _l_* ( *v*) with *ā _l_* = *η _l_* *N _l_*r*_l_*q (in pA/ *µ*m ^2^), and

$$\;\varphi_{l}(v)=\exp\left[b_{l}\left(\frac{\eta_{l}v-v_{l}}{v_{\text{T}}}\right)\right]-\exp\left[(b_{l}-1)\left(\frac{\eta_{l}v-v_{l}}{v_{\text{T}}}\right)\right],\;(33)$$

where *v _l_/η _l_* represents the reversal potential for the *l*th current, *l* ∈ {1, ..., *M*}. There is experimental evidence for some ion channels that supports the replacement of *η _l_* *r _l_*q for a constant ( Nonner & Eisenberg, 1998). For instance, it is reasonable to assume that the single channel current for many types of Na ^+^channels is 1 pA ( Aldrich *et al.* (1983)). Assuming that the change in charge density with respect to voltage (typically referred to as membrane capacitance in conductance-based models) is a constant *C _m_* (pF/ *μ*m ^2^, Herrera-Valdez (2020)), the time-dependent change in transmembrane potential can then be written as

$$\;\partial_{t}v=-\sum_{l=1}^{M}a_{l}p_{l}\varphi_{l}(v),\;(34)$$

with *v* in mV and *a _l_* = *ā* *_l_*/ *C _M_* in mV/mS (pA/pF) represents the normalized current amplitude for the *l*th transport mechanism, *l* ∈ (1, ..., *N*). Note that only electrogenic transport mechanisms are included.

### Cardiac pacemaking in the sinoatrial node

**Figure 4.**
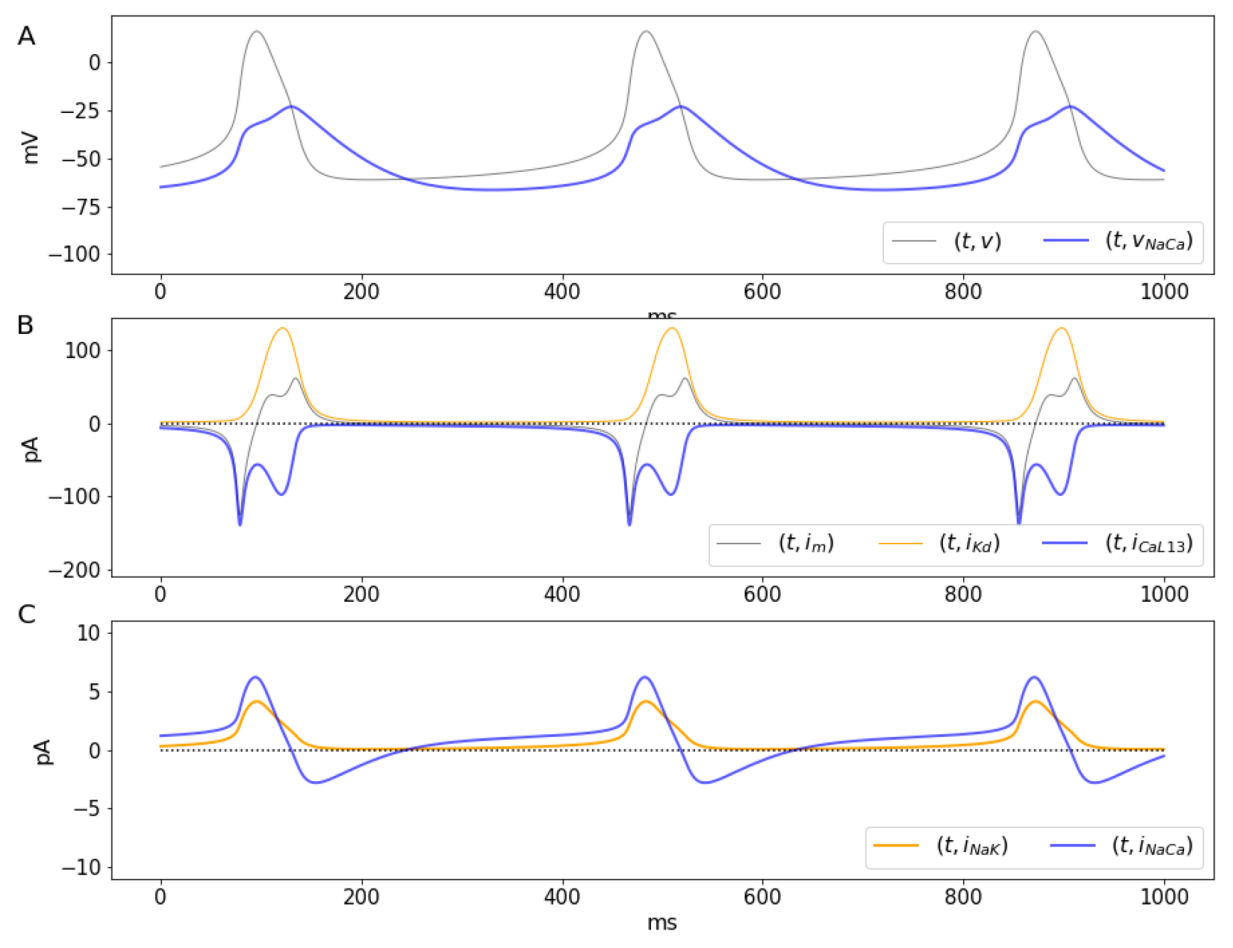
Central sinoatrial node pacemaking dynamics using the system ( 35)–( 42). **A**. Transmembrane potential (gray) and the reversal potential *v*_NaCa_ (blue) as a function of time. **B**. Dynamics of large calcium (blue) and potassium (orange) currents in pA. The total current shown by a gray line labelled as *i _m_*. **C**. Currents mediated by NaK ATPases (orange) and Na-Ca exchangers (blue), respectively. Notice the carrier mediated currents ( **C**) are about one order of magnitude smaller than those mediated by channels shown ( **B**).

The pacemaking dynamics of cells in the rabbit sinoatrial node ( Figure 4) can be modelled as low dimensional dynamical systems based on the assumption that *v* changes as a function of a combination of channel-mediated electrodiffusion and pumping mechanisms involving Ca ^2+^, K ^+^, and Na ^+^ ( Herrera-Valdez & Lega, 2011; Herrera-Valdez, 2014). Explicitly, assume that Ca ^2+^ transport is mediated by L-type Ca ^2+^ channels ( Mangoni *et al*., 2003) and Na ^+^-Ca ^2+^ exchangers ( Sanders *et al*., 2006). K ^+^ transport is mediated by delayed-rectifier voltage-activated channels ( Shibasaki, 1987), and Na ^+^-K ^+^ ATPases ( Herrera-Valdez & Lega, 2011; Herrera-Valdez, 2014).

The temporal evolution for *v* can then be described by

$$\;\partial_{t}v\;=\;–J_{\text{NaK}}\;(v)\;–J_{\text{NaCa}}(v,c)\;–\;J_{\text{CaL}}(v,w,c)\;–\;J_{\text{KD}}(v,w),\;(35)$$

where *w* represents the proportion of activated K ^+^ channels (and also, the proportion of inactivated Ca ^2+^ channels) ( Av-Ron *et al*., 1991; Herrera-Valdez & Lega, 2011). The variable *c* represents the intracellular Ca ^2+^ concentration. The transmembrane currents (normalised by the membrane capacitance), are given by

$$\;J_{\text{NaK}}\;(v)\;=\;a_{\text{NaK}}\;\varphi_{\text{NaK}}(v),\;(36)$$

$$\;J_{\text{NaCa}}\;(v,c)\;=\;a_{\text{NaCa}}\;\varphi_{\text{NaCa}}(v,c),\;(37)$$

$$\;J_{\text{KD}}\;(v,w)\;=\;a_{\text{KD}}\;w\;\varphi_{\text{KD}}(v),\;(38)$$

$$\;J_{\text{CaL}}\;(v,w,c)\;=\;a_{\text{Ca}}\;(1\;–\;w)F_{m}(v)\;\varphi_{\text{Ca}}(v,\;c),\;(39)$$

where *φ _x_* is a difference of exponential functions as defined in Equation (16), with *x* ∈ {NaK, NaCa, KD, CaL}.

The steady state for the activation of voltage-dependent channels in the model is described by a generic function of the form

$$\;F_{u}(v)=\frac{exp\left(g_{u}\frac{v-v_{u}}{v_{\text{T}}}\right)}{1+exp\left(g_{u}\frac{v-v_{u}}{v_{\text{T}}}\right)},\;u\in \left\{m,w\right\}\;(40)$$

with labels *m* and *w* used for L-type Ca ^2+^ and delayed rectifier K ^+^ channels, respectively. *F _u_* is increasing as a function of *v*, with a graph of sigmoidal shape ( Herrera-Valdez & Lega, 2011). The parameters *g _u_* and *v _u_* represent the steepness and the half-activation potential, respectively. The voltage-dependent activation for the L-type Ca ^2+^ channels ( Equation (39)) is assumed to be fast (quasi-stedy state), so it is described by the function *F _m_*( *v*) ( Av-Ron *et al.,* 1991; Herrera-Valdez & Lega, 2011; Rinzel & Ermentrout, 1989).

The activation of K ^+^ currents recorded in voltage-clamp experiments often displays sigmoidal time courses that resemble logistic population dynamics ( Covarrubias *et al*., 1991; Hodgkin & Huxley, 1952; Tsunoda & Salkoff, 1995). The dynamics of the proportion of activated K ^+^ channels, *w*, evolve according to

$$\;\partial_{t}w\;=\;w^{k_{w}}[F_{w}(v)\;–\;w]R_{w}(v),\;(41)$$

where *F _w_* and *R _w_* respectively representing the voltage-dependent steady state and rate (1/ms) for the opening of K *_d_* channels ( Willms *et al*., 1999).

The rate of activation for K ^+^ channels is a voltage-dependent function of the form

$$\;R_{w}(v)=r_{w}\left[exp\left(b_{w}g_{w}\frac{v-v_{w}}{v_{\text{T}}}\right)+exp\left(\text{(}b_{w}-1\text{)}g_{w}\frac{v-v_{w}}{v_{\text{T}}}\right)\right],\;(42)$$

where *b _w_* represents a bias in the conformational change underlying channel activation. The function *R _w_* has the shape of a hyperbolic cosine when *b _w_* is 1/2.

During pacemaking oscillations, the intracellular Ca ^2+^ concentration may change 10-fold or more ( Rasmusson *et al*., 1990a; Rasmusson *et al*., 1990b). Therefore, the system includes an equation for the dynamics for *c*, assuming linear convergence to a steady state *c*_∞_ in the absence of Ca ^2+^ fluxes, and an increase proportional to the total transport of Ca ^2+^ ions through L-type channels and Na ^+^-Ca ^2+^ exchangers ( Figure 4). Explicitly,

$$\;\partial_{t}c\;=\;r_{c}\;(c_{\infty }\;–\;c)\;–k_{c}[J_{\text{CaL}}(v,w,c)\;–\;J_{\text{NaCa}}(v,c)],\;(43)$$

where *k _c_* ( *µ*M/mV) represents the impact of the transmembrane Ca ^2+^ fluxes on the intracellular Ca ^2+^ concentration. The term *−r _c_ c* can be thought of as a generic form of buffering and other transport mechanisms contributing to decrease the intracellular Ca ^2+^ concentration. The minus sign in front of *k _c_* accounts for the fact that the sign of the *J*_CaL_ is negative. The sign in front of *J*_NaCa_ is because the forward flux of Ca ^2+^ mediated by the Na-Ca exchanger is opposite to that of electrodiffusive Ca ^2+^. The transmembrane concentrations of Na ^+^ and K ^+^ across the membrane are assumed to change negligibly ( Herrera-Valdez & Lega, 2011; Rasmusson *et al*., 1990a; Rasmusson *et al*., 1990b).

The solutions of Equations (35)– (43) with parameters as in Table 3 reproduce important features of the membrane dynamics observed in the rabbit’s central sinoatrial node, including the period ( *ca.* 400 ms), amplitude ( *ca.* 70 mV), and the maximum *∂ _t_v* (<10 V/s) of the action potentials ( Zhang *et al*., 2000).

**Table 3.** Parameters for the cardiac SAN pacemaker model. The amplitudes *a*_*l*_ can be thought of as *ā*_*l*_/ *C*_*M*_ where *C*_*M*_ is a constant that represents the rate of change in charge around the membrane as a function of *v*, and *l* ∈ {Ca *L*, K, NaK, NaCa}.

Parameter	Value	Units	Description
*C _M_*	30	pF	Membrane capacitance
*ā*_Ca_	1	pA	Amplitude for the L-type Ca ^2+^ current
*ā*_K_	100	pA	Amplitude for the K ^+^ current
*ā*_NaK_	1	pA	Amplitude for the Na ^+^-K ^+^ current
*ā*_NaCa_	3	pA	Amplitude for the Na ^+^-Ca ^2+^ current
*a*_Ca_ = *ā*_Ca_ */C _M_*	0.0333	pA/pF	Amplitude for the normalized L-type Ca ^2+^ current
*a*_K_ = *ā*_K_ */C _M_*	3.3333	pA/pF	Amplitude for the normalized K ^+^ current
*a*_NaK_ = *ā*_NaK_ */C _M_*	0.03333	pA/pF	Amplitude for the normalized Na ^+^-K ^+^ current
*a*_NaCa_ = *ā*_NaCa_ */C _M_*	0.1	pA/pF	Amplitude for the normalized Na ^+^-Ca ^2+^ current
*v*_ATP_	-420	mV	Potential ATP hydrolysis
*v*_Na_	60	mV	Nernst potential for Na ^+^
*v*_K_	-89	mV	Nernst potential for K ^+^
*v*_NaK_ = 3 *v _Na_ −* 2 *v*_K_ + *v*_ATP_	-62	mV	Reversal potential for the for Na ^+^-K ^+^ ATPase current
*v*_NaCa_ = 2 *v*_Ca_ − 3 *v*_Na_	–	mV	Reversal potential for the for Na ^+^-Ca ^2+^ current ( *v*_Ca_ depends continuously on [ *Ca*] _*i*_)
*v*_*m*13_	-25	mV	Half-activation potential for Ca _*v*13_ L-type Ca ^2+^-current ( Mangoni *et al.,* 2003)
*v*_*w*_	-25	mV	Half-activation potential for the transient K ^+^-current ( Shibasaki, 1987)
*g*_*m*13_	5	–	Activation slope factor for the Ca _*v*13_ L-type Ca ^2+^-current
*g*_*w*_	3.6	–	Activation slope factor for the K ^+^-current ( Shibasaki, 1987)
*r*_*w*_	0.005	s ^−1^	Activation rate for the cardiocyte K ^+^-current
*k*_*w*_	0.3	−	Exponent for the K ^+^ -activation variable
*b*_*w*_	0.35	–	Activation slope factor for the K ^+^-current
*b*_NaK_	0.35	–	Non-rectification bias for the Na ^+^-K ^+^-current
*b*_K_	0.1	–	Rectification for the transient K ^+^-current ( Shibasaki, 1987)
*b*_Na_	0.5	–	Non-rectification bias for the transient Na ^+^-current
*b*_Ca_	0.5	–	Non-rectification bias for the Ca _*v*13_ L-type Ca ^2+^-current
*c*_*∞*_	0.1	*µ*M	Minimal (resting) intracellular Ca ^2+^- concentration
*r*_*c*_	0.02	ms ^−1^	Ca ^2+^ removal rate
*k*_*c*_	0.00554	–	Conversion factor between Ca ^2+^ current and intracellular Ca ^2+^ concentration

**Figure 5.**
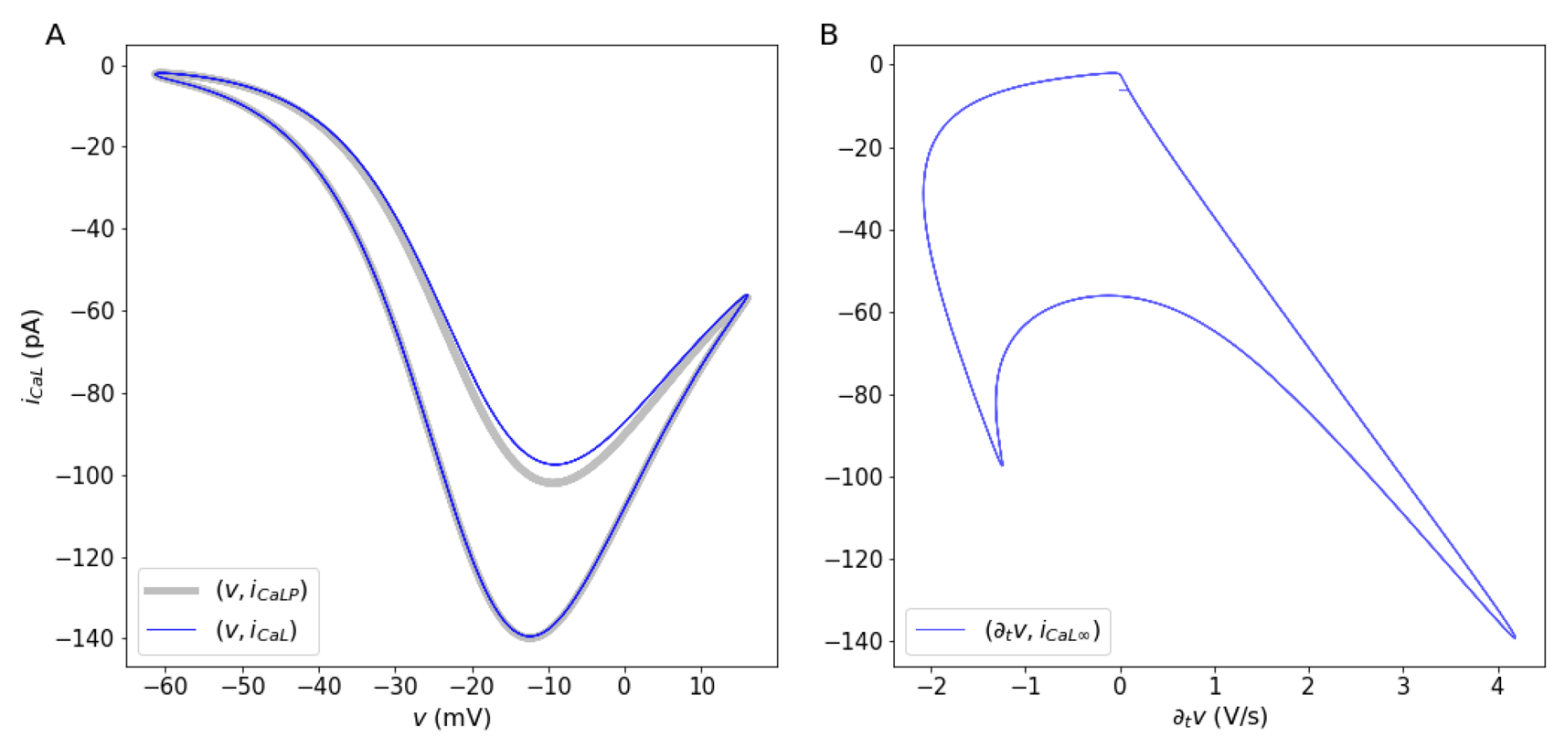
Dynamics of the calcium current and double activation during pacemaking oscillations. **A**. Behaviour of the inactivating L-type Ca ^2+^ current with respect to the transmembrane potential (blue line) and a non-inactivating current (gray line). Notice that two points with locally maximum current amplitude occur during the action potential. The local minimum with larger current values occurs during the upstroke of the action potential. The local minimum with smaller current values occurs during the downstroke of the action potentials. ( **B**) Calcium current as a function of the time-dependent change in *v*. The maximum rate of change for *v* occurs when the calcium current reaches its maximum amplitude.

A number of interesting features of ionic fluxes can be observed from closer examination of the solutions of Equation (35)– Equation (43). First, the Na-Ca current reverses during pacemaking, as *v* = *v*_NaCa_ ( Figure 4A, blue line). Between the initial depolarisation and until the maximum downstroke rate, approximately, *v*_NaCa_ *< v*, which means that *J*_NaCa_ *>* 0. Then, Ca ^2+^ extrusion by the Na-Ca exchanger occurs only for a brief period of time during the downstroke and also after each action potential ( Figure 4C, blue line). Second, as previously reported in different studies involving spiking dynamics, the time course of the Ca ^2+^ current shows a partial inactivation with a double peak ( Figure 4B, blue line) around a local minimum ( Carter & Bean, 2009; Rasmusson *et al*., 1990a; Rasmusson *et al*., 1990b), and in agreement with data from voltage-clamp experiments ( Mangoni *et al*., 2006). A number of models have been constructed in attempts to reproduce the double activation by making extra assumptions about gating ( Rasmusson *et al*., 1990a; Rasmusson *et al*., 1990b). For instance, some models include a second activation variable, or multiple terms in the steady state gating, or in the time constant for activation or inactivation. However, the explanation for the double peak can be much simpler. The calcium current *J*_CaL_ is a negative-valued, non monotonic function for *v < v*_Ca_, which can be thought of as a product of a amplitude term that includes gating and the function *φ*_CaL_. The normalised current *J*_CaL_ has a local minimum (maximum current amplitude) around -10 mV ( Figure 4B, blue line and Figure 5A, blue line), after which the current decreases, reaching a local maximum as the total current passes through zero, at the peak of the action potential around 10 mV ( Figure 4B, where *∂ _t_ v* = 0). The first peak for the Ca ^2+^ current occurs when *v* reaches the maximum depolarisation rate ( Figure 5B). As *v* increases (e.g. upstroke of the action potential). The second peak for the current occurs as the membrane potential decreases, and passes again through the region where the maximal current occurs (local minimum for *J _Ca_*). The two local minima for *J*_CaL_ occur at different amplitudes because of the difference in the evolution of *v* during the upstroke and the downstroke of the action potential ( Figure 5A, blue line, and Figure 5B, where *∂ _t_ v* = 0). It is important to remark that the dual role played by *w* is not the cause of the double activation. This is illustrated by analysing the behaviour of a non-inactivating *J*_CaL_ without the inactivation component ( Figure 5A, gray line). The double activation can also be observed in models in which the activation of K ^+^ channels and the inactivation of Ca ^2+^ or Na ^+^ channels are represented by different variables ( Rasmusson *et al*., 1990a) and in dynamic voltage clamp experiments on neurons in which there are transient and persistent sodium channels ( Carter & Bean, 2009).

**Figure 6.**
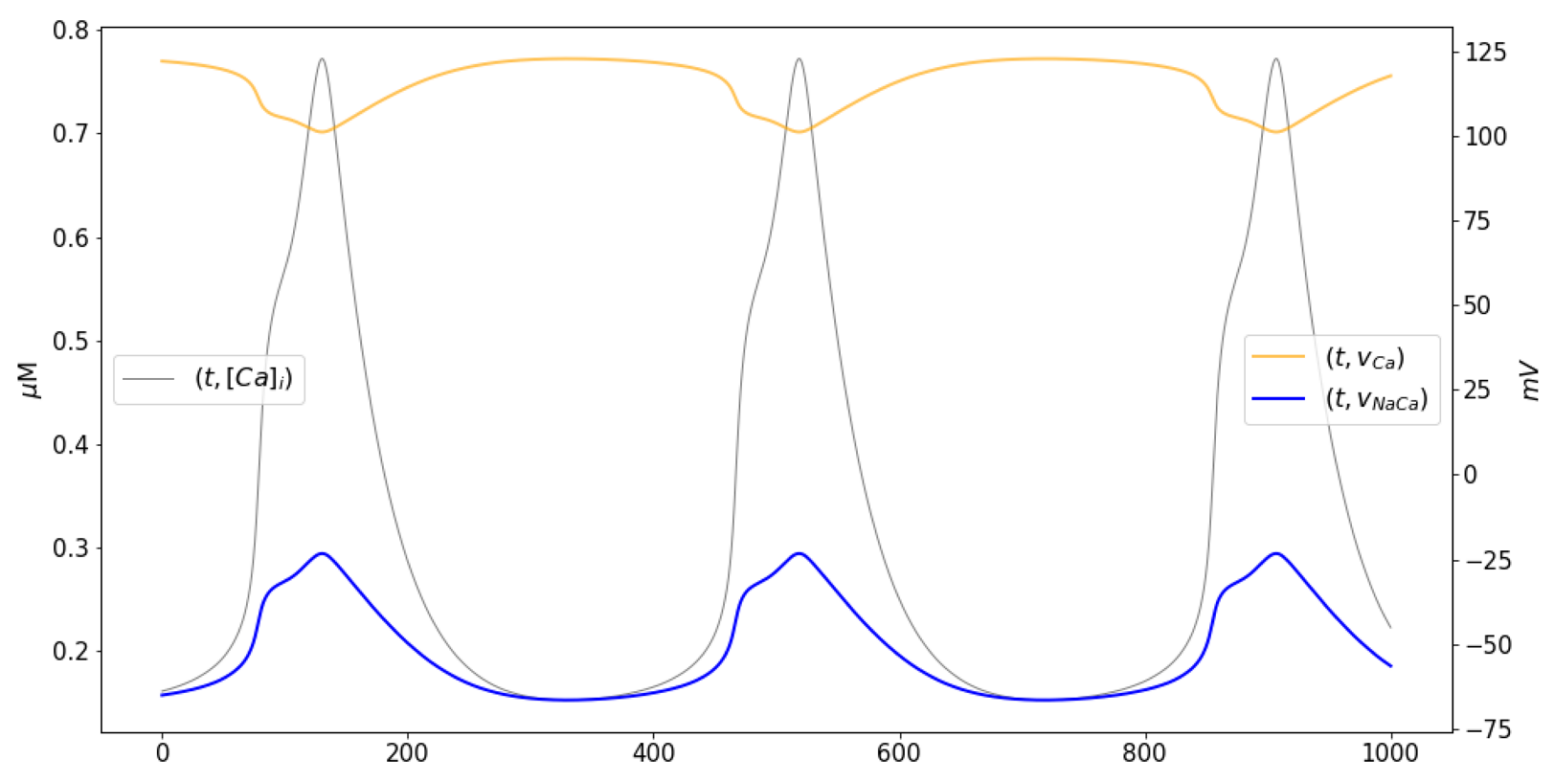
Calcium dynamics during pacemaking. Time courses of the intracellular calcium concentration (gray, left axis), the Nernst potential for Ca ^2+^ (orange, right axis), and the reversal potential for the Na-Ca exchanger (blue, right axis). Notice the two phases of calcium increase that occur in agreement with the double peak observed in the calcium current (see Figure 4B, blue trace).

The double peak in the Ca ^2+^ current reflects on the intracellular Ca ^2+^ concentration ( Figure 6, gray line), and by extension, on the Nernst potential for Ca ^2+^ ( Figure 6, blue line), which display two increasing phases and two decreasing phases, respectively. The first and faster phase in both cases occur during the initial activation of the L-type channels. The second phase occurs during the downstroke, as second peak of the Ca ^2+^ current occurs. As a consequence, the reversal potential for the Na-Ca exchanger, *v*_NaCa_ = 2 *v*_Ca_ *−* 3 *v*_Na_ ( Figure 6, orange line) also has two phases, this time increasing. Increasing the intracellular Ca ^2+^ ( Figure 6, gray line) concentration decreases the Nernst potential for Ca ^2+^, and vice versa. By extension, *v*_NaCa_, becomes larger when *c* increases. Ca ^2+^ enters the cell in exchange for Na ^+^ that moves out when *v > v*_NaCa_, during most of the increasing phase and the initial depolarisation phase of the action potential (blue lines in Figure 4A and C, and Figure 6).

### Fast spiking interneuron dynamics

To construct of a simple model for the dynamics of a fast spiking (FS) striatal interneuron, assume the transmembrane potential depends on three currents respectively mediated by Na-K pumps, non-inactivating K ^+ ^channels, and Na ^+ ^channels with transient dynamics, with voltage-dependent gating in both channels. It is also assumed that the proportion of activated K ^+ ^is represented by a variable *w* ∈ [0, 1], which also represents the proportion of inactivated Na ^+ ^channels ( Av-Ron *et al.,* 1991; Rinzel, 1985). That is, 1 − *w* represents the proportion of non-inactivated Na ^+ ^channels. Since *w* models the activation of a *population* of channels, it makes sense assume that its dynamics follow a logistic scheme, without adding extra powers to *w*. This also follows the experimentally observed dynamics which have been reported repeatedly, including those of delayed-rectifier K ^+ ^currents recorded in voltage clamp (see for instance Figure 3 in Hodgkin & Huxley, 1952).

Explicitly, the dynamics for the FS-interneuron membrane can be described by a system with two coupled differential equations of the form

$$\partial_{t}v=-(1-w)F_{m}(v)a_{\text{Na}T}\;\psi_{\text{NaT}}(v)-wa_{\text{KaD}}\psi_{\text{KaD}}(v)-a_{\text{NaK}}\;\psi_{\text{NaK}}(v),\;(44)$$

$$\partial_{t}w=w^{k_{w}}[F_{w}(v)-w]R_{w}(v),\;(45)$$

The activation rate for K ^+ ^channels depends is a voltage-dependent functions *R _w_* and *F _w_* as defined for the cardiac pacemaking model. It is also assumed that the activation of sodium channels is at a quasi steady state as a function of *v*.

**Figure 7.**
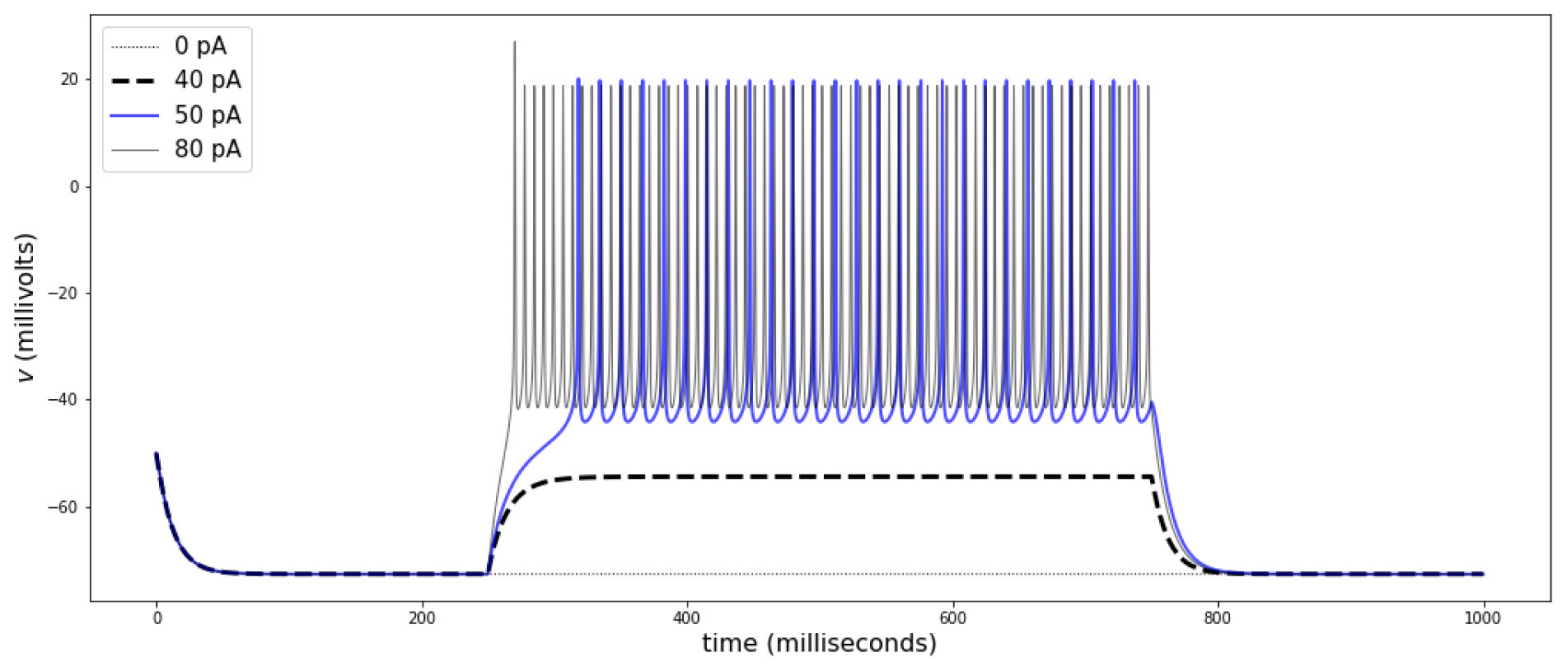
Rest to spiking transitions of FS interneuron under current clamp. The traces show responses to current-clamp stimulation of different amplitudes. The transition between rest and spiking with a rheobase occurs between 40 and 50 pA, as shown for some FS neurons in the mouse striatum ( Orduz *et al.,* 2013). The traces correspond to stimulation amplitudes of 0 (gray dots), 40 (black dashed line), 50 (blue), and 80 pA (gray). Parameters can be found in Table 4.

Striatal FS interneurons display maximum *∂ _t_v* between 100 and 200 V/s. In current clamp mode, most neurons are silent, and show transitions between rest and repetitive spiking at a rheobase current of approximately 90 pA, with initial firing rates between 50 and 60 Hz and a delay to first spike in the transition that decreases as the stimulus amplitude increases ( Figure 7, parameters in Table 4).

**Table 4.** Parameters for the fast spiking interneuron model.

Parameter	Value	Units	Description
Current amplitudes and capacitance for the neuronal membrane model			
*C _m_*	30	pF	Membrane capacitance
*ā*_NaK_	67	pA	Amplitude for the Na ^+^-K ^+^ ATPase current
*ā*_K_	4400	pA	Amplitude for the delayed-rectifier K ^+^ current
*ā*_Na_	1400	pA	Amplitude for the transient Na ^+^ current
*v*_ATP_	-430	mV	Potential ATP hydrolysis
*v*_NaK_ = 3 *v _Na_*–2 *v*_K_ + *v*_ATP_	-72	mV	Reversal potential for the for Na ^+^-K ^+^ ATPase current
*v*_K_	-89	mV	Nernst potential for K ^+^
*v*_Na_	60	mV	Nernst potential for Na ^+^
*v _mT_*	-17	mV	Half-activation potential for the transient Na ^+^-current
*v*_w_	-5	mV	Half-activation potential for the transient K ^+^-current
*g _mT_*	5	–	Activation slope factor for the transient Na ^+^-current
*g _w_*	4	–	Activation slope factor for the K ^+^-current
*r _w_*	2	*s* ^–1^	Activation rate for the neuronal K ^+^-current
*k _w_*	1	–	Activation exponent for the K ^+^-current
*b _w_*	0.3	–	Activation slope factor for the K ^+^-current
*b*_NaK_	0.5	–	Non-rectification for the Na ^+^-K ^+^-current
*b*_K_	0.5	–	Non-rectification for the transient K ^+^-current
*b*_Na_	0.5	–	Non-rectification for the transient Na ^+^-current

To include these properties into the model, the membrane capacitance was specified first, then the maximum *∂ _t_v* was adjusted by fitting the parameter *a*_NaT_, and then the contributions for the K ^+ ^channels and the Na-K ATPase are set to obtain spiking and fit the rheobase. The model in Equations (44)– (45) reproduces dynamics observable in fast spiking neurons in CA1 ( Erisir *et al.,* 1999) or in the striatum ( Orduz *et al.,* 2013; Tepper *et al.,* 2010).

Even though FS-interneuron spiking is conditional to the reception of input, the dynamics of the system can be thought of as qualitatively similar to those observe in the SAN. Within the time of a single spike, and by extension during the repetitive spiking regime, if *v* increases, *w* also increases, but at a slower rate in comparison to *v*. This is because the activation *w* is always moving toward its steady state value, which increases as *v* increases. Once *w* increases, the Na ^+ ^current tends to decrease and the K ^+ ^current tends to increase, thereby causing a decrease in *v*. The slower dynamics in *w* relative to those in *v* capture the delay between the amplification caused by the Na ^+ ^current and the recovery caused by the negative feedback of the K ^+ ^current. The current mediated by Na/K-ATPase acts as an extra attracting force toward *v*_NaK _that increases nonlinearly as the distance between *v* and *v*_NaK _increases.

## Discussion

A general, macroscopic model for transmembrane fluxes has been derived by directly calculating the work required to transport molecules across the membrane. The derivation is based on a general thermodynamic scheme that takes into account the rate, stoichiometry, and the direction in which the molecules are transported across the membrane. These biophysical parameters are then combined to write expressions for directional fluxes based on van’t Hoff (1884) and Arrhenius (1889) formulations, weighted as in the Butler/Erdey-Gruz/Volmer equation ( Butler, 1924; Erdey-Grúz & Volmer, 1930). The result is a general description of the transmembrane molecular flux as a difference of exponential functions ( Equation (16)) that describes the transport dynamics of molecules in the "forward" and "backward" directions, relative to a source compartment. This general description describes transport due to electrodiffusion mediated by channels, and also translocation mediated by pumps ( Table 1). The two exponential functions depend on a common expression involving the transmembrane concentrations of the molecules being transported, and possibly the transmembrane potential when transport is electrogenic.

Rectification, an asymmetry in the flow during transport, is typically modelled modifying the dynamics of the gating variables for the current. The general formulas for transmembrane transport include a bias term *b* that controls the relative contribution of inward and outward fluxes the transport. Hence, different types of rectification can be described by favouring one of the directions for transport, conceptually in line with the "anomalous rectification" originally reported by Katz (1949) for K ^+^ in muscle cells. The bias term is *not* part of any gating mechanism. Instead, it represents the asymmetry in bidirectional flux. For instance, in K ^+^ channels, the inward (outward, respectively) rectification occurs when the fourth transmembrane segment of the channel (S4) is located closer to the intracellular (extracellular) portion of the membrane in its open configuration ( Riedelsberger *et al*., 2015). There are other reports that show that asymmetries in bidirectional transport occur as a consequence of changes in the three dimensional structure of the protein mediating the transport ( Halliday & Resnick, 1981; Quistgaard *et al*., 2013). Therefore, the rectification term can be thought of as representing a structural component of the transmembrane protein through which molecules move ( Figure (3)). Outward rectification in K ^+^ channels can be explained, for instance, by biasing the flux of K ^+^ the forward (outward) direction ( *b*_K_ *>* 1 */*2). Instead, inward rectification can be obtained by biasing the transport in the backward (inward) direction ( *b*_K_ < 1 */*2). It is important to remark that non-rectifying currents with *b* = 1 */*2 are nonlinear functions of ∆ *G*, which shows that the nonlinearity of the current-voltage relationships is not the defining characteristic of rectification; as argued in some textbooks (see Kew & Davies, 2010).

The formulation for transmembrane flux may be rewritten in different alternative forms that can be found throughout the literature (see Equation (11) and Equation (14), Goldman, 1943; Johnston *et al*., 1995). Of particular interest, the widely used conductance-based models for current from the seminal work of Hodgkin & Huxley (1952) turn out to be linear approximations of the general current described here ( Herrera-Valdez, 2012; Herrera-Valdez, 2014). This explains why the Hodgkin & Huxley (1952) model captures many of the defining features of action potential generation, in spite of modelling ionic currents as if they were resistive. Another interesting case is that electrodiffusive transmembrane currents derived from the Nernst-Planck equation ( Nernst, 1888; Planck, 1890), turn out particular cases of the general formulation presented here (see also Herrera-Valdez, 2014, for details). Examples include the constant field approximation ( Clay *et al*., 2008; Hille, 1992; Johnston *et al*., 1995), the non-rectifying currents proposed by Endresen *et al*. (2000), and more general electrodiffusive currents that includes a bias term accounting for rectification ( Herrera-Valdez, 2014; Johnston *et al*., 1995).

Possibly of interest to mathematicians working on bifurcation theory, a third order approximation ( Equation (17)), can be used to construct models resembling the Fitz-Hugh system ( FitzHugh, 1955; FitzHugh, 1961; Fitz-Hugh, 1966), which yield very close approximations to the full model, while keeping biophysical characteristics of real systems like rectification and specific ionic permeabilities, and the multiplicative interaction between the slow variable *w* and the fast variable *v*; properties that the Fitz-Hugh polynomials do not have. Third order approximations also open the possibility of expanding on the analysis of dynamical systems based on these general formulas to study normal forms and bifurcations. Depending on the ions involved in each transmembrane transport mechanism, the third order approximations for current can be shown to be very close to the full function in Equation (16) ( Figure 2). Another possible use of the third order approximations could be to construct network models that take into account nonlinearities included in the general formulation, but at a reduced computational cost in comparison to the full model. This possibility is currently being tested and will be reported in the near future. A similar comparison has been made between the full model and the conductance-based approximation taking a dynamical systems perspective and also by means of computational simulations in Herrera-Valdez (2012).

One question of interest because of its possible impact on the interpretation of results from existing modelling studies is how do the excitability and the resulting dynamics in a model of membrane dynamics change when using the thermodynamic transmembrane currents or their approximations? The question has been addressed in a study in which two simple neuronal models with currents mediated by Na ^+^ and K ^+^, each equipped with the same biophysical gating properties and the same relative contributions for the currents, but one with currents as in Equation (22), the other with conductance-based currents. The two models display a number of qualitative and quantitative differences worth considering while making the choice of a model in theoretical studies ( Herrera-Valdez, 2012). For a start, the two models are not topologically equivalent across many ratios of the relative contributions of K ^+^ and Na ^+^ channels ( Herrera-Valdez, 2012); as would be expected by the fact that conductance-based formulations are only linear approximations of the general currents, around the reversal potential for each current. One of the most notable differences between the general formulation and the conductance-based formula is the contribution of the nonlinear, high order terms from Equation (16), which results in more realistic upstrokes for action potentials and an overall increased excitability; in this case characterised in terms of the minimum sustained current necessary to produce at least one action potential ( Herrera-Valdez, 2012). The increased excitability of the membrane with the general formulation is due, in part, to the large, exponential contribution of the open Na ^+^ and Ca ^2+^ channels, but not the K ^+^ channels, to the change in the transmembrane potential near rest. The time course of the Na ^+^ current during the beginning of the action potential with the general model is much sharper than that of the conductance-based formulation, resulting in a faster upstroke of the action potential; and in better agreement with observations in cortex and other tissues ( Naundorf *et al*., 2006). It is important to remark that the sharper increase in the change of the membrane potential shown using the general formulation is a consequence of the nonlinear driving force terms of the current in the general model (the flux term in the general formulation), and not in the activation dynamics for the transient Na ^+^ current. The nonlinearities unravelled by the general formulation could thus be part of the explanation for the observed sharpness at the beginning of the action potential upstroke observed in different experiments. However, such nonlinearities do not rule out other contributions, such as cooperation between Na channels, or the effects of spatial differences in Na-channel densities, as pointed out by Brette (2013) for the case of action potentials in cortical pyramidal cells. In the models presented here, the sharpness in the upstrokes of neuronal action potentials combine nonlinear factors including the flux given by the general formulation, channel densities, and gating that does not follow linear, but logistic-like dynamics.

The general formulation for both passive and active transmembrane transport can be thought of as a tool that facilitates the construction and analysis of models of membrane potential dynamics. The generality and versatility of the thermodynamic transmembrane transport formulations is illustrated with models of cardiac pacemaking interneuron fast spiking. The ion fluxes in the model are assumed to be mediated by two different types of voltage-gated channels and two different types of pumps, all represented with the *same* functional form (see DiFrancesco & Noble (1985); Herrera-Valdez & Lega (2011); Rasmusson *et al*. (1990b) for examples in which that is not the case).

One important advantage of the general formulation is that it includes the possibility of explicitly estimating the number of channels or pumps mediating each of the transport mechanisms of interest. This has proven to be useful to study the relative contributions of different currents to the excitability of neurons (see Herrera-Valdez *et al*., 2013) and cardiocytes ( Herrera-Valdez, 2014).

Another extension of possible interest is that of modelling the transmembrane transport between organelles and the cytosolic compartment, which can be done by directly replacing the difference *c _s_ − d _s_* with -1 or 1, in Equation (1), accounting for the direction of transmembrane motion of molecules relative to the outer compartment. This and other generalisations enable the possibility of studying the interdependence between electrical excitability across tissues and animal species ( Herrera-Valdez *et al*., 2013), and its cross-interactions with metabolism and other processes of physiological importance, all from a general theoretical framework with common formulations.

**Implications for experimentalists.** One of the main advantages of the general expressions is that fits to ionic currents can be made straight from the voltage-clamp data without much effort, and without having to calculate conductances, which amounts to imposing the assumption that the current-voltage relationship is linear. Fits to experimental currents can then be directly put into equations describing the change in the membrane potential, and model membrane dynamics of interest without having to make many extra adjustments, as it is the case for most conductance-based models restricted to data.

The model for current in Equation (22) has been used to construct simplified models for the membrane dynamics of different cell types using experimental data. Examples include fast spiking interneurons in the mice striatum ( Figure 7), motor neurons in *Drosophila melanogaster* ( Herrera-Valdez *et al*., 2013), pyramidal cells in the young and ageing hippocampus of rats ( McKiernan *et al*., 2015), medium spiny neurons in the mouse striatum ( Suárez *et al*., 2015), rabbit sinoatrial node cells ( Herrera-Valdez, 2014), and other types of excitable cells ( McKiernan & Herrera-Valdez, 2012).

## Conclusions

A general model that describes physiological transmembrane transport of molecules has been derived by considering basic thermodynamical principles. The model unifies descriptions of transport mediated by channels and pumps, it can model biases in either one of the directions of flow, and it can be easily converted into a model for current in the case of electrogenic transport. As it is desirable in all models, the general expressions can be thought of as extensions of some previous models. In particular, it is shown that the conductance-based model for current turns out to be a first order approximation of the general formulation.

The general formulation presented here can be used to build general models of phenomena involving transmembrane transport using a unified framework ( Shou *et al*., 2015).

## Data availability

All data underlying the results are available as part of the article and no additional source data are required to reproduce the results presented here.

## Footnotes

1 The transmembrane potential for which there is a zero net flux of s-ions across the membrane, as given by the Nernst-Planck equation, is

$$v_{s}=\frac{\text{kT}}{z_{s}\text{q}}\ln\left(\frac{{\left[s\right]}_{0}}{{\left[s\right]}_{1}}\right).$$

2 The change in Gibbs free energy in "standard" conditions of ATP, ADP and P *_i_* at concentrations of 1M and a water concentration of approximately 55M, yields $\text{Δ}G_{\text{ATP}}^{0}$ between -28 and -34 kJ/mol, ( Bergman *et al*., 2010)). The second term in Equation (6) depends on a reaction quotient [ADP] [P *_i_*] */*[ATP], which explains deviations of ∆ *G* from the standard change.
