# Supplementary material for: A thermodynamic description for physiological transmembrane transport
Source: F1000Res. 2021 May 19;7:1468. Originally published 2018 Sep 14. [Version 3] doi: 10.12688/f1000research.16169.3 (PMC6259595; doi:10.12688/f1000research.16169.3)
Supplement: Peer review history

# Peer review: Reviewer response for version 2

**DOI:** 10.5256/f1000research.18524.r40941

**Published:** 2018-11-22

**Authors:** Kyle C.A. Wedgwood

**Affiliations:** 1 Living Systems Institute, College of Engineering, Maths and Physical Sciences, Centre for Biomedical Modelling and Analysis, University of Exeter, Exeter, UK

I am pleased to report that the author has done a great job in addressing all of my (and reviewer 2's) comments.

 I therefore now recommend this article for indexing without further modification.

Is the work clearly and accurately presented and does it cite the current literature?

Yes

If applicable, is the statistical analysis and its interpretation appropriate?

Not applicable

Are all the source data underlying the results available to ensure full reproducibility?

Partly

Is the study design appropriate and is the work technically sound?

Yes

Are the conclusions drawn adequately supported by the results?

Yes

Are sufficient details of methods and analysis provided to allow replication by others?

Yes

Reviewer Expertise:

NA

I confirm that I have read this submission and believe that I have an appropriate level of expertise to confirm that it is of an acceptable scientific standard.

# Peer review: Reviewer response for version 1

**DOI:** 10.5256/f1000research.17657.r38997

**Published:** 2018-10-30

**Authors:** Moisés Santillán

**Affiliations:** 1 Moisés Santillán's Quantitative Biology & Complex Systems Lab, Center for Research and Advanced Studies of the National Polytechnic Institute (CINVESTAV), Monterrey, Nuevo León, Mexico

I found this paper quite interesting. It introduces a mathematical formulation for ion transport across cell membranes, based in thermodynamic principles. The author further shows that other, more common formulations, are special cases of the more general one introduced here. Finally, the author employs the introduced formulation to study the dynamics of SA pacemaker cells, and is able to explain the experimentally-observed double activation of calcium channels.

 Overall, I have a good opinion of this paper and recommend its indexing. There are only a few minor points that should be addressed:

 1. Page 3, first column, beginning of last paragraph: Eq. (10) is referred to before being introduced.

 2. In my opinion, Eq. (8) is not self-evident, and its origin should be explained.

 3. Same thing for Eq. (33).

 4. The procedure to estimate the parameter values in Table 2 must be explained. Moreover, a parameter sensitivity analysis should be performed to assess the robustness of the obtained results.

Is the work clearly and accurately presented and does it cite the current literature?

Yes

If applicable, is the statistical analysis and its interpretation appropriate?

Not applicable

Are all the source data underlying the results available to ensure full reproducibility?

No source data required

Is the study design appropriate and is the work technically sound?

Yes

Are the conclusions drawn adequately supported by the results?

Yes

Are sufficient details of methods and analysis provided to allow replication by others?

Yes

Reviewer Expertise:

NA

I confirm that I have read this submission and believe that I have an appropriate level of expertise to confirm that it is of an acceptable scientific standard.

# Response

**Published:** 2018-11-03

**Authors:** Marco Arieli Herrera-Valdez

**Affiliations:** Universidad Nacional Autónoma de México, Mexico

***The answers to comments made by reviewers are written in italics after each comment.***

***Answers to comments from reviewer 2***

Overall, I have a good opinion of this paper and recommend its indexing. There are only a few minor points that should be addressed:

1. Page 3, first column, beginning of last paragraph: Eq. (10) is referred to before being introduced.

*Corrected the error in the cross reference.*

2. In my opinion, Eq. (8) is not self-evident, and its origin should be explained.

*Added a reference pointing to a Boltzmann distribution.*

3. Same thing for Eq. (33).

*Added a reference for the work of Willms et al., 1999.*

4. The procedure to estimate the parameter values in Table 2 must be explained. Moreover, a parameter sensitivity analysis should be performed to assess the robustness of the obtained results.

*The reviewer points to a very important issue. There are different publications and books addressing the robustness of conductance-based models (which are similar to the current one), from a dynamical systems perspective, using bifurcation theory. That being said, I agree that the parameter sensitivity analysis should be done for completeness, as this is a different model, but that analysis is beyond the scope of this paper. Nevertheless, parameter sensitivity analysis is addressed in a publication currently in preparation. In that publication, I address a few issues, including whether the gating formulas yield correct predictions in consideration of the work of Hodgkin and Huxley and the powers used for the fits on voltage clamps, and whether the relationships between parameters are correct, as a consequence of the modeling using those powers. It is worth mentioning that the parameters are in line with the experimentally measured magnitudes for cardiocytes and neurons, as those being modeled.*

# Peer review: Reviewer response for version 1

**DOI:** 10.5256/f1000research.17657.r38363

**Published:** 2018-10-01

**Authors:** Kyle C.A. Wedgwood

**Affiliations:** 1 Living Systems Institute, College of Engineering, Maths and Physical Sciences, Centre for Biomedical Modelling and Analysis, University of Exeter, Exeter, UK

Thank you for inviting me to review the article entitled "A thermodynamic description for physiological transmembrane transport". This article presents a unifying mathematical formulation for passive and active transport across plasma membranes. This is achieved by considering the energy required to achieve transport of the molecules. For active transport, in which molecules are transported against their concentration gradient, the required energy is positive. In contrast, passive movement down a concentration gradient does not require energy transfer. In previous modelling frameworks, active and passive transport have been described via distinct models for pumps and channels respectively. The major contribution of this paper is to combine these models into one formulation.

 After presenting the initial mathematical formulation, the article proceeds to make comparisons with existing literature and demonstrates that earlier mathematical models of channels are special cases of the more general framework. The article ends with an example in which the full model can explain a double activation event of Ca ^2+^ channels, which cannot be done so satisfactorily by existing models.

 In general, the article is well-written, with arguments made concisely and coherently. Aside from a few points which require further clarification, I believe that this article is sufficiently rigorous and of interest to the community to warrant publication. In particular, channel and pump models have long been used in the mathematical study of excitability in cell membranes. These models have been used to support and guide experimental studies as well as advancing the applied mathematics field in its own right. As such, improvements to these models should be welcomed by the field.

 I will now address specific issues with the article:

1. In general, I believe that the figure captions could do with expanding to guide the reader's eyes to the features of the plot that they should be focusing on.
2. p2, first paragraph: At present, the discussion of passive and active transport in the Introduction is a little confusing. In particular, there are transmembrane proteins that undergo conformational changes to transport molecules down their concentration gradient and channels that achieve this by effectively forming pores through the membrane (so that further conformational change is not needed to effect transport). Furthermore, the reference Blicher & Heimberg, 2013 reports on spontaneous (protein-free) formation of channels in synthetic membranes. Whilst this is an interesting topic, it should be made clearer how this relates to physiological membranes. Finally, it might also be useful to the reader to give an example of how some of the different pump configurations work (e.g. uniporter, symporter) to facilitate understanding of where energy transfer is required.
3. p2, 3rd paragraph: "Examples of fits to..." This is not a sentence. Please revise.
4. p2, 3rd paragraph: The author should highlight what "noncentral issues" they are referring to.
5. p2, 5th paragraph, minor: It would be useful to highlight that c _s_, d _s_ \in {0,1}.
6. p2, Eq. (1): v is not defined.
7. p2, footnote, minor: Change "log" to "ln" here for consistency with Eq. (1) and (3).
8. p3, 2nd paragraph: It would be good to clarify in this first sentence the distinction between \Delta G and \Delta G_s (or avoid using the former entirely).
9. p3, 2nd paragraph: S = {Na,H} typo here.
10. p3, Eq. (5): Similarly to point (7), it is not clear what \Delta G represents here.
11. p3, Eq. (6): Terms in this equation need defining properly in several regards. Firstly, \Delta G _ATP_^0 ^should be defined. Secondly, it should be made clear that concentrations are intracellular (as opposed to in Eq. (1) (3), which correspond to both extra- and intracellular compartments). This is particularly confusing for P _i _in which the subscript could easily be misread as being an intracellular label. Finally, it is not clear how the equality \Delta GA _TP_ = qv _ATP _holds. Perhaps, as in footnote (1), it could be made clear that this relies on the definition of the reversal potential for ATP.
12. p3, Eq. (6): I would also here like to take the opportunity to raise a point about the general framework. The models here account for the energy required to move molecules with and against their concentration gradients. In cases of active transport, as indicated by Eq. (10), ATP is then hydrolysed to meet the energy deficit required to power the pump. This then means that the concentration of ATP, relative to ADP is then changing. Indeed, at maximal rates of exercise, the ATP concentration in skeletal muscle can fall by up to 20% of its initial value. Moreover, in certain cell types, local ratios between ATP and ADP affect the opening and closing of ion channels (e.g. K _ATP_). Eq. (10) implies that ATP and ADP in the cell are constant, in spite of the necessity that it must change. One must, therefore, conclude that an assumption is being made that ATP changes are small relative to the total amount of ATP. This assumption should be stated, or a discussion around how changes to ATP concentrations effect Eq. (10) should be made.
13. p3, final paragraph of first column: Wrong equation being referred to.
14. p3, Eq. (9): A physical interpretation for parameters r and b should be given. Additionally, and referring back to my previous comment (1), the text here discusses the relative magnitudes of r for electrodiffusive and active transport. Thus, this point should make clear that ion channels should be faster than both facilitated diffusion through carrier proteins and active transport via pumps.
15.  p4, Eq. (11): For consistency with Eq. (5), 'Extra' in the subscripts should read 'Ext'. Also, it should be noted that v _Extra_ should be the v _ATP_ from Eq. (6).
16. p4, "In particular, for electrodiffusive (passive) transport of ions of type l": It is not clear what the author is referring to here (or how it is different from what is written in Eq. (15)).
17. p4, "The first, more complex form...": Does the author mean to reference Eq. (11) here?
18. p4, After Eq. (16), minor: "Ampere" needs capitalising.
19. p5, Table 1: Is v _0_ here the same as in Eq. (15)? If so, the dependency on v _Ext_ seems to have disappeared.
20. p6, Fig. 2: What is plotted on the ordinate axis in this graph? It appears to have units of pA, but the text says that this is a plot of alpha/beta, which should be dimensionless.
21. p8, "Electrodiffusive transport": I'm not really sure what the point of this section is since the results presented here have mostly been mentioned previously in this manuscript.
22. p8, after Eq. (31): Is the N written here supposed to be N_l? Also, there is a typo after Eq. (31) (with -> where).
23. p9, Eq. (33): m is not defined.
24. p9, Eq. (35-39): Change symbol for current to i _x_ for consistency with previous equations.
25. p9, after Eq. (39): Presumably, the author here means that the intracellular Ca2+ concentration changes (rather than the extracellular). This should be clarified. Also, the text below Eq. (40) appears to imply a distinction between free and bound Ca ^2+^, but there is no buffering process described in the model, which may cause confusion.
26. p9, Fig. 2: Please state what i _m_ and i _CaL13_ are in these plots.
27. p10, Table 2: The units of \bar{a} are inconsistent with the main text.
28. p12, "Another possible use of the third order approximations is in the construction of network models.": Please could the author state what they mean here?
29. p13, discussion on action potential shape: I was wondering if the author could here compare their argument surrounding the shape of the action potential near initiation with those put forward here (Brette, 2013 ^1^), which puts forward a case for multi-compartment models to describe the sharpness of the spike.
30. SI1, after (A3): There appears to be a reference missing here.
31. SI2: Headers are required in these data files so that the values herein can be related to the physical quantities they represent.
32. It would be useful for the author to upload any code used to generate the plots in the article.

Is the work clearly and accurately presented and does it cite the current literature?

Yes

If applicable, is the statistical analysis and its interpretation appropriate?

Not applicable

Are all the source data underlying the results available to ensure full reproducibility?

Partly

Is the study design appropriate and is the work technically sound?

Yes

Are the conclusions drawn adequately supported by the results?

Yes

Are sufficient details of methods and analysis provided to allow replication by others?

Yes

Reviewer Expertise:

NA

I confirm that I have read this submission and believe that I have an appropriate level of expertise to confirm that it is of an acceptable scientific standard, however I have significant reservations, as outlined above.

## References

[1] : Sharpness of spike initiation in neurons explained by compartmentalization. *PLoS Comput Biol*.2013;9(12) : 10.1371/journal.pcbi.1003338 e1003338 10.1371/journal.pcbi.1003338 24339755PMC3854010

# Response

**Published:** 2018-11-02

**Authors:** Marco Arieli Herrera-Valdez

**Affiliations:** Universidad Nacional Autónoma de México, Mexico

***The answers to comments made by reviewers are written in italics after each comment.***

***Answers to comments from Reviewer 1***

Thank you for inviting me to review the article entitled "A thermodynamic description for physiological transmembrane transport". This article presents a unifying mathematical formulation for passive and active transport across plasma membranes. This is achieved by considering the energy required to achieve transport of the molecules. For active transport, in which molecules are transported against their concentration gradient, the required energy is positive. In contrast, passive movement down a concentration gradient does not require energy transfer. In previous modelling frameworks, active and passive transport have been described via distinct models for pumps and channels respectively. The major contribution of this paper is to combine these models into one formulation.

After presenting the initial mathematical formulation, the article proceeds to make comparisons with existing literature and demonstrates that earlier mathematical models of channels are special cases of the more general framework. The article ends with an example in which the full model can explain a double activation event of Ca2+ channels, which cannot be done so satisfactorily by existing models.

In general, the article is well-written, with arguments made concisely and coherently. Aside from a few points which require further clarification, I believe that this article is sufficiently rigorous and of interest to the community to warrant publication. In particular, channel and pump models have long been used in the mathematical study of excitability in cell membranes. These models have been used to support and guide experimental studies as well as advancing the applied mathematics field in its own right. As such, improvements to these models should be welcomed by the field.

Thank you for your comments. They make the article clearer and better.. Please find answers to each of them below.

I will now address specific issues with the article:

1. In general, I believe that the figure captions could do with expanding to guide the reader's eyes to the features of the plot that they should be focusing on.

*I have added phrases pointing to specific parts of the graphs or mentioning specific things to notice in each of the figures.*

2. p2, first paragraph: At present, the discussion of passive and active transport in the Introduction is a little confusing. In particular, there are transmembrane proteins that undergo conformational changes to transport molecules down their concentration gradient and channels that achieve this by effectively forming pores through the membrane (so that further conformational change is not needed to effect transport). Furthermore, the reference Blicher & Heimberg, 2013 reports on spontaneous (protein-free) formation of channels in synthetic membranes. Whilst this is an interesting topic, it should be made clearer how this relates to physiological membranes. Finally, it might also be useful to the reader to give an example of how some of the different pump configurations work (e.g. uniporter, symporter) to facilitate understanding of where energy transfer is required.

*The first paragraph in p2 has been rewritten taking into account the above observations.*

*3. *p2, 3rd paragraph: "Examples of fits to..." This is not a sentence. Please revise.

*Thank you, corrected.*

4. p2, 3rd paragraph: The author should highlight what "noncentral issues" they are referring to.

*Rewrote the paragraph for clarity and punctuality.*

5. p2, 5th paragraph, minor: It would be useful to highlight that cs, ds \in {0,1}.

*Highlighted*

6. p2, Eq. (1): v is not defined.

*Added definition*

7. p2, footnote, minor: Change "log" to "ln" here for consistency with Eq. (1) and (3).

*Changed log to ln*

8. p3, 2nd paragraph: It would be good to clarify in this first sentence the distinction between \Delta G and \Delta G_s (or avoid using the former entirely).

*Corrected.*

9. p3, 2nd paragraph: S = {Na,H} typo here.

*Corrected.*

10. p3, Eq. (5): Similarly to point (7), it is not clear what \Delta G represents here.

*Added a phrase to explain it.*

11. p3, Eq. (6): Terms in this equation need defining properly in several regards. Firstly, \Delta GATP0 should be defined. Secondly, it should be made clear that concentrations are intracellular (as opposed to in Eq. (1) (3), which correspond to both extra- and intracellular compartments). This is particularly confusing for Pi in which the subscript could easily be misread as being an intracellular label. Finally, it is not clear how the equality \Delta GATP= qvATP holds. Perhaps, as in footnote (1), it could be made clear that this relies on the definition of the reversal potential for ATP.

*Added square brackets and subindices to indicate that the intracellular concentrations of ADP, ATP, and Pi are intracellular. Also corrected a typo, there was an extra q in the term for $\Delta G_0$*

12. p3, Eq. (6): I would also here like to take the opportunity to raise a point about the general framework. The models here account for the energy required to move molecules with and against their concentration gradients. In cases of active transport, as indicated by Eq. (10), ATP is then hydrolysed to meet the energy deficit required to power the pump. This then means that the concentration of ATP, relative to ADP is then changing. Indeed, at maximal rates of exercise, the ATP concentration in skeletal muscle can fall by up to 20% of its initial value. Moreover, in certain cell types, local ratios between ATP and ADP affect the opening and closing of ion channels (e.g. KATP). Eq. (10) implies that ATP and ADP in the cell are constant, in spite of the necessity that it must change. One must, therefore, conclude that an assumption is being made that ATP changes are small relative to the total amount of ATP. This assumption should be stated, or a discussion around how changes to ATP concentrations effect Eq. (10) should be made.

*Added a comment about the assumptions and a comment about the possibility of changes in concentration for ATP, ADP, and Pi*

13. p3, final paragraph of first column: Wrong equation being referred to.

*Corrected cross reference*

14. p3, Eq. (9): A physical interpretation for parameters r and b should be given. Additionally, and referring back to my previous comment (1), the text here discusses the relative magnitudes of r for electrodiffusive and active transport. Thus, this point should make clear that ion channels should be faster than both facilitated diffusion through carrier proteins and active transport via pumps.

*Added clarification and phrases providing interpretation for the two parameters.*

15. p4, Eq. (11): For consistency with Eq. (5), 'Extra' in the subscripts should read 'Ext'. Also, it should be noted that vExtra should be the vATP from Eq. (6).

*Added clarification and deleted the “ra” in the extra suffix.*

16. p4, "In particular, for electrodiffusive (passive) transport of ions of type l": It is not clear what the author is referring to here (or how it is different from what is written in Eq. (15)).

*Rephrased the sentence for correction.*

17. p4, "The first, more complex form...": Does the author mean to reference Eq. (11) here?

*Moved the sentence up to the paragraph with Eq. (11) and added the cross reference*

18. p4, After Eq. (16), minor: "Ampere" needs capitalising.

*Corrected*

19. p5, Table 1: Is v0 here the same as in Eq. (15)? If so, the dependency on vExt seems to have disappeared.

*vExt is not written in those cases where it is zero.*

20. p6, Fig. 2: What is plotted on the ordinate axis in this graph? It appears to have units of pA, but the text says that this is a plot of alpha/beta, which should be dimensionless.

*Corrected text.*

21. p8, "Electrodiffusive transport": I'm not really sure what the point of this section is since the results presented here have mostly been mentioned previously in this manuscript.

*Added sentences making the point more explicit and took away the sentence in previous paragraphs where the single ion energy was discussed to avoid repetition.*

22. p8, after Eq. (31): Is the N written here supposed to be N_l? Also, there is a typo after Eq. (31) (with -> where).

*Changed N for M to avoid confusion.*

23. p9, Eq. (33): m is not defined.

*Changed the order in which equations are presented and rephrased so that variables and parameters are better explained*

24. p9, Eq. (35-39): Change symbol for current to ix for consistency with previous equations.

*The currents in equations 35-39 are normalized by the membrane capacitance. Therefore, to avoid abuse of notation, added a note remarking the change in the letter for the functions, explaining it is due to normalization.*

25. p9, after Eq. (39): Presumably, the author here means that the intracellular Ca2+ concentration changes (rather than the extracellular). This should be clarified. Also, the text below Eq. (40) appears to imply a distinction between free and bound Ca2+, but there is no buffering process described in the model, which may cause confusion.

*Added sentences to clarify these points.*

26. p9, Fig. 2: Please state what im and iCaL13 are in these plots.im represents the total current.

*Added a sentence to the figure caption.*

27. p10, Table 2: The units of \bar{a} are inconsistent with the main text.

*Corrected by deleting the bars in the table for normalized amplitude entries. \bar{a} is in pA, a = \bar{a}/Cm in pA/pF*

28. p12, "Another possible use of the third order approximations is in the construction of network models.": Please could the author state what they mean here?

*Expanded the sentence to clarify the point. The idea is to use cubic approximations to build network models taking advantage of the possible reduction in computational cost. However, as is explained in the paragraph, cubic approximations need to be tested first to address possible issues that may arise from using approximations.*

29. p13, discussion on action potential shape: I was wondering if the author could here compare their argument surrounding the shape of the action potential near initiation with those put forward here (Brette, 2013 1), which puts forward a case for multi-compartment models to describe the sharpness of the spike.Added a comment about the results in the paper by Brette, 2013.

*Thank you for pointing it out. Added a reference to his work too.*

30. SI1, after (A3): There appears to be a reference missing here.

*Edited the reference, it is for Equation 31.*

31. SI2: Headers are required in these data files so that the values herein can be related to the physical quantities they represent.

*Edited data files to include headers.*

32. It would be useful for the author to upload any code used to generate the plots in the article.

*Uploaded the code in an jupyter notebook.*
